# Transcriptome-level assessment of the impact of deformed wing virus on honey bee larvae

**DOI:** 10.1038/s41598-021-94641-3

**Published:** 2021-07-22

**Authors:** Zih-Ting Chang, Yu-Feng Huang, Yue-Wen Chen, Ming-Ren Yen, Po-Ya Hsu, Tzu-Han Chen, Yi-Hsuan Li, Kuo-Ping Chiu, Yu-Shin Nai

**Affiliations:** 1 Department of Biotechnology and Animal Science, National Ilan University, Yilan City, 260 Taiwan; 2 Genomics Research Center, Academia Sinica, Taipei City, 115 Taiwan; 3 Department of Entomology, National Chung Hsing University, Taichung City, 402 Taiwan

**Keywords:** Microbiology, Entomology

## Abstract

Deformed wing virus (DWV) prevalence is high in honey bee (*Apis mellifera*) populations. The virus infects honey bees through vertical and horizontal transmission, leading to behavioural changes, wing deformity, and early mortality. To better understand the impacts of viral infection in the larval stage of honey bees, artificially reared honey bee larvae were infected with DWV (1.55 × 10^10^ copies/per larva). No significant mortality occurred in infected honey bee larvae, while the survival rates decreased significantly at the pupal stage. Examination of DWV replication revealed that viral replication began at 2 days post inoculation (d.p.i.), increased dramatically to 4 d.p.i., and then continuously increased in the pupal stage. To better understand the impact of DWV on the larval stage, DWV-infected and control groups were subjected to transcriptomic analysis at 4 d.p.i. Two hundred fifty-five differentially expressed genes (DEGs) (fold change ≥ 2 or ≤ -2) were identified. Of these DEGs, 168 genes were downregulated, and 87 genes were upregulated. Gene Ontology (GO) analysis showed that 141 DEGs (55.3%) were categorized into molecular functions, cellular components and biological processes. One hundred eleven genes (38 upregulated and 73 downregulated) were annotated by KO (KEGG Orthology) pathway mapping and involved metabolic pathways, biosynthesis of secondary metabolites and glycine, serine and threonine metabolism pathways. Validation of DEGs was performed, and the related gene expression levels showed a similar tendency to the DEG predictions at 4 d.p.i.; cell wall integrity and stress response component 1 (*wsc1*), *cuticular protein* and *myo-inositol 2-dehydrogenase* (*iolG*) were significantly upregulated, and *small conductance calcium-activated potassium channel protein* (*SK*) was significantly downregulated at 4 d.p.i. Related gene expression levels at different d.p.i. revealed that these DEGs were significantly regulated from the larval stage to the pupal stage, indicating the potential impacts of gene expression levels from the larval to the pupal stages. Taken together, DWV infection in the honey bee larval stage potentially influences the gene expression levels from larvae to pupae and reduces the survival rate of the pupal stage. This information emphasizes the consequences of DWV prevalence in honey bee larvae for apiculture.

## Introduction

Honey bees, *Apies mellifera*, play an essential role in pollination and provide many high-value products. The number of colonies has increased over the past half-century^1^. Collectively, honey bees contribute more than USD 215 billion annually to global agriculture and ecosystem services^2–5^. However, honey bee populations are often subjected to diseases and environmental changes (e.g*.*, climatic stress), and reductions have been observed. Since 2006, colony collapse disorder (CCD) has caused catastrophic losses of honey bee colonies in the North American and European apiculture industries^3,6,7^. An approximately 23% loss of honey bee colonies was estimated in the overwinter in 2006–2007, with a 36% loss in 2007–2008 in the United States^6,8^. More than 2.4 million colonies were affected during that period^6,9^. Therefore, worldwide concern is increasing regarding honey bee colony losses, particularly in countries that are developing/promoting agriculture.

Several factors related to honey bee colony losses have been proposed and validated; one of the most crucial is honey bee pathogens^6,10,11^. Pathogens of honey bees can be classified into parasites, fungi, bacteria, and viruses. Based on metagenomics, viruses, microsporidia (*Nosema ceranae* and *Nosema apis*), mites (*Varroa destructor*) and parasite infections are commonly found in honey bee colonies with honey bee colony losses^8,12^. Among these pathogens, Israeli acute paralysis virus (IAPV) was the major factor causing large damage to agriculture during 2006–2007 in the United States^8^. A previous study suggested that infection by IAPV may promote the spread of other viruses, and *Varroa* mites carrying IAPV can also increase the prevalence of other honey bee viruses^13,14^. Therefore, the diseases caused by honey bee viruses are difficult to diagnose, control and recover.

To date, 24 honey bee viruses have been recognized^15,16^, and most are positive-sense ( +), single-stranded RNA (ssRNA) viruses, including acute bee paralysis virus (ABPV), black queen cell virus (BQCV), chronic bee paralysis virus (CBPV), deformed wing virus (DWV), Israeli acute paralysis virus (IAPV), sacbrood virus (SBV), and *Varroa destructor* virus-1 (VDV-1)^12,15,17^. Among these viruses, DWV is the most prevalent in honey bees. More than 20% of adult honey bees were reported to be DWV carriers in one bee hive, regardless of the strength of the honey bees^18^. DWV has also been listed as a pathogen of emerging infectious diseases (EIDs) and has become a threat to wild bee species (family *Apidae*) because of the overlapping living areas of wild bee species. Additionally, the bee-infecting virus can be transmitted from wild bees to reared honey bees, causing viral cross infection^19^. Furthermore, honey bee-infecting viruses may infect a wide range of insects^20^.

DWV is a ( +) ssRNA virus belonging to the *Iflavirida*e family. It is a nonenveloped icosahedral virus approximately 30 nm in diameter, and the genome length is 10,140 nt^21^. The DWV species complex comprises three master variants (DWV-A, -B and -C type)^22,23^. The obvious symptom of emerged adult bees with DWV infection is a crippled-wing appearance. The transmission route of DWV includes either vertical or horizontal transmission; vertical transmission is typically from the ovary of the viral carrier queen or testis of drones, while horizontal transmission is caused by faecal-oral transmission or *Varroa* mites (*V. destructor*)^24,25^. The *Varroa* mite plays an important role in DWV transmission. *A. mellifera* may be more susceptible to transmission through mites and results in DWV predominating in colonies, although DWV cannot propagate in the mites^20,26–29^.

Many studies have focused on the deformed wing symptoms or learning behaviour of adult bees, which are caused by DWV transmission through *Varroa* mites^17,30,31^. Regarding gene expression, the expression levels of the relish gene and antimicrobial defensin gene showed a correlation with the DWV-Varroa complex and antiviral defence mechanism^32^. Additionally, infection with DWV suppresses the upstream Toll pathway^33^. Several mechanisms, including autophagy, endocytosis, melanization, and JAK/STAT (Janus kinase/signal transducer and activator of transcription), Toll, JNK (c-Jun N-terminal kinase), RNA interference (RNAi), and MAPK (mitogen-activated protein kinase) pathways, play roles in the antiviral defence of honey bees^16^. The pathological impacts of viruses on honey bees are governed by the intricate balance between host defence and virus counterdefence mechanisms^16,34^. To better understand the impacts on DWV infection in the larval stage and continuous impacts on the development process, we infected honey bee larvae with DWV using an artificial larval feeding system. The infected larvae were collected and then subjected to transcriptomic analysis for differentially expressed gene (DEG) identification. Based on our analysis, genes with higher fold changes (fc ≥ 4 or ≤ -4) were validated, and these genes were upregulated or downregulated continuously during the DWV infection process to the pupal stage. This study aimed to describe the continuous effects on DWV infection from the larval stage to the pupal stage and provide information for the risk evaluation of honey bee viral diseases.

## Materials and methods

### Artificial rearing of *A. mellifera* larvae

Honey bee larvae were collected from healthy honey bee (*A. mellifera*) colonies of NIU apiaries (National Ilan University, Taiwan). Healthy honey bee colonies were defined as containing nine frames of the hive with ca. 25,000 workers and a normal spawning queen. More than 300 1-day-old larvae were collected into 24-well culture plates (10 larvae/per well) containing a basic larval diet (BLD)^35^. The method for collecting 1-day-old larvae followed the protocol proposed by Ko et al.^36^. The artificial rearing method was modified based on methods proposed by Hanley et al. and Ko et al.^36,37^. The 1-day-old larvae were reared at 34–35 °C and 95% relative humidity in freshly prepared BLD. The BLD was changed daily for 3 days. The larvae were then transferred to new 24-well culture plates (5 larvae/per well) containing freshly prepared BLD for the DWV infection experiment.

### Screening of DWV-carrying honey bee samples

All honey bee colonies used in this study were screened for DWV and six other common honey bee virus infections—ABPV, BQCV, CBPV, IAPV, SBV and *Varroa destructor* virus-1 (VDV-1)—by reverse transcription polymerase chain reaction (RT-PCR) as described below. Midgut tissue was collected from three honey bees in 500 µL of 0.1% phosphate buffered saline (PBS) solution as one sample and homogenized with a homogenizing pestle. Total RNA was extracted from 200 µL of homogenized midgut tissue using an RNA Isolation Kit (FairBiotech, TW), and the remaining part was stored at − 80℃. The extracted RNA (1 μg) was then treated with DNase I at 25 °C for 15 min and inactivated by EDTA at 65 °C for 10 min. DNase I-treated total RNA was reverse transcribed using a GScript RTase kit (GeneDireX, USA) following the manufacturer’s instructions. The reaction was incubated at 42 °C for 1.5 h. and then terminated at 70 °C for 15 min. RT-PCR was performed using eight honey bee viral gene-specific primer sets (Supplementary Table 1). PCR amplification was performed as follows using a Primus 96 Plus Thermal Cycler (MWG-Biotech, DE): an initial preheating step at 94 °C for 5 min, and then 30 cycles at 95 °C for 30 s, 50 °C for 30 s, and 72 °C for 1 min, followed by a 5-min final extension at 72 °C and storage at 20 °C. The PCR product was analysed by electrophoresis on a 2% agarose gel in 1 × TAE buffer. Ten DWV-positive samples without other viral signals were collected and subjected to quantification of viral RNA copies.

### Quantification of DWV and viral infection based on a plasmid standard curve

The selected DWV-positive samples were subjected to real-time quantitative polymerase chain reaction (RT-qPCR) to calculate the viral copy number. For RT-qPCR, partial DWV gene fragments were amplified by PCR and then cloned using a T&A clone kit (RBC Bioscience, TW); the ligated plasmid DNAs were transformed into *Escherichia coli* DH5α (RBC Bioscience, TW). Bacterial colonies of the expected recombinant plasmids were chosen for further analysis by commercial sequencing. The sequence-confirmed plasmids were then used to generate a qPCR standard curve (Supplementary Fig. 1). Undetermined DWV samples were diluted tenfold and tested using Applied Biosystems StepOne Real-time PCR systems with DyNAmo ColorFlash SYBR Green qPCR reagents (Thermo Scientific, USA). The qPCR programme was set as follows: 95 °C for 1 min and 40 cycles of 95 °C for 15 s and 60 °C for 1 min. The results for serially diluted standard plasmids were used to construct a standard curve. The linear standard equation for DWV quantification was generated by plotting the crossing point (Cp) versus the log_10_ of the initial plasmid copy number as follows: y = -3.3831x + 45. 306, R^2^ = 0.9992. The range of the assay was from 10^4^ to 10^10^ copies per sample. Therefore, the viral genome copy numbers of undetermined DWV samples were calculated based on the formula for the plasmid standard curve. The quantitated samples were used to infect honey bee larvae.

### DWV infection of honey bee larvae

The larvae of the honey bee colonies were detected for the prevalence of 7 viruses before DWV infection as described above (Supplementary Table 1; Supplementary Fig. 2). Honey bee colonies without other identified viral signals were used for viral infection. One-day-old larvae were collected and divided into control (noninfected) and infected groups (approximately 150 larvae for each) and then reared as mentioned above. Each honey bee larva was inoculated in 60 µl of 0.1% PBS solution containing 1.55 × 10^10^ DWV copies, while the control group was inoculated in 60 µl of 0.1% PBS solution only. After the larvae consumed the PBS solution for 12 h, fresh BLD was added to the larva. All experiments were performed in triplicate. The survival rates of the infected and control groups were observed and recorded daily until pupation. The survival analysis was performed using SPSS (IBM SPSS Statistics version 20).

### Detection of viral copies after DWV infection

To determine the viral copy number, three DWV-infected larvae were collected as one sample and in triplicate at 0 (the larvae infected with DWV at 3 h. post infection), 2, 4 and 9 d.p.i. (3-, 5-, 7- and 12-day-old larvae). After RNA extraction, conventional RT-PCR and quantitative analysis of DWV were performed as described above using the DWV primer set (Supplementary Table 1).

### Preparation of RNA samples for next-generation sequencing

The honey bee larvae were collected at 4 d.p.i. (7-day-old larvae) and homogenized using Tissue Lyser II (QIAGEN, DE) at f = 30/s for 1 min, and this procedure was repeated three times. Total RNA was extracted using TRIzol reagent (Invitrogen Life Technologies, USA) following the manufacturer’s instructions. The extracted RNA sample was then purified using the RNeasy Mini Kit (QIAGEN, DE). The RNA purity and integrity were checked using a Nanodrop 1000 Spectrophotometer V3.5 (Thermo Scientific, USA) and Qubit 2.0 Fluorometer (Invitrogen, USA). The total RNA samples from three DWV-infected larvae were pooled as one replicate, and two biological replicates were used for library construction. The size distribution of the RNA samples was determined using the Fragment Analyzer system (Agilent, USA). The RNA samples were then used to construct a library for transcriptome sequencing.

### Next-generation sequencing and raw data processing

The mRNA from the DWV-infected and noninfected larvae was further purified for sequencing library construction using an TruSeq stranded mRNA Library Prep Kit (Illumina, San Diego, CA, USA) according to the manufacturer's protocol. Finally, the DNA fragments were selectively enriched by PCR and then qualified and quantified using the Fragment Analyzer system (Agilent, USA) and qPCR, respectively, for DNA library quality control analysis. These two samples were sequenced in parallel using an Illumina NovaSeq 6000 sequencer to generate high-throughput transcriptome sequencing data with a read length of 151 bp by paired-end (PE) technology. The sequencing adaptors were trimmed from raw PE reads using Trimmomatic^38^, and then read quality checking from trimmed PE reads was performed using PRINseq^39^. The PE reads were also mapped to the genomes of 7 viruses to screen for viral infections.

### Differentially expressed gene (DEG) analysis

The quality PE reads were mapped to the honey bee genome index (*A. mellifera* Amel_HAv3.1) using HISAT2^40^ with corresponding gene annotation information (GCF_003254395.2_Amel_HAv3.1_genomic.gff). The read counts of genes were computed using HTSeq^41^. To exclude genes with extremely low expression in the analysis, the most expressed replicate in either the control or DWV-infected sample must have had a minimum of 10 reads (after normalizing for sequencing depth). The read counts were then subjected to differentially expressed gene (DEG) analysis using edgeR^42^ (Supplementary Fig. 3). Multiple testing errors were corrected using the false discovery rate (FDR). Statistical analysis of fold change was performed, and a significant difference in the expression of these two groups was defined as an FDR < 0.05. Additionally, we considered differentially expressed genes as having a log2 ratio ≥ 1 or ≤ -1 (fold change ≥ 2 or ≤ -2). To facilitate logarithmic transformation, a value of 1 was added to the CPM value of the remaining genes. The gene expression heat map was then generated using the R package^43^. DEGs were further subjected to GO enrichment analysis (http://geneontology.org/page/go-enrichment-analysis) and KEGG Automatic Annotation Server (KAAS, http://www.genome.jp/tools/kaas/) analysis for gene orthologue assignment and pathway mapping^44–46^.

### qRT-PCR validation

To validate the results of DEG analysis, four upregulated [*wsc1* (LOC100577331), *cuticular protein* (LOC724464), *iolG* (LOC552024) and *GAPDH* (LOC413924)] and three downregulated genes [*CYP6A1* (LOC413908), *SK* (LOC410317) and *MINPP1* (LOC409751)] were selected and subjected to quantitative RT-qPCR analysis at different d.p.i. Primer Express v3.0 was applied to design primer sets of target genes for RT-qPCR analysis (Supplementary Table 2). The RNA of the DWV-infected and control groups at 0 (larvae infected with DWV at 3 h. post infection), 2, 4, 6 and 9 d.p.i. was extracted as described above (three larvae were pooled as one sample). Before performing RT-qPCR, the RNA was treated with DNase I (Invitrogen Life Technologies, USA) following the manufacturer’s instructions to reduce genomic DNA contamination. The DNase I-treated total RNA samples were reverse transcribed using the GScript RTase Kit (GeneDireX, USA) following the manufacturer’s instructions. The reaction mixture was incubated at 42 °C for 1.5 h., and then the reaction was terminated at 70 °C for 15 min. Real-time qPCR was performed using a Thermo Scientific Verso SYBR Green 1-step qRT-PCR ROX Mix Kit (Thermo Scientific, USA) in a 96-well Bio-Rad CFX96 Real-Time PCR System (Bio-Rad, USA). All reactions were performed in five replicates. The relative gene expression levels were calculated using the 2^−△△Ct^ method^47^.

## Results and discussion

### DWV infection in honey bee larvae

**Figure 1:**
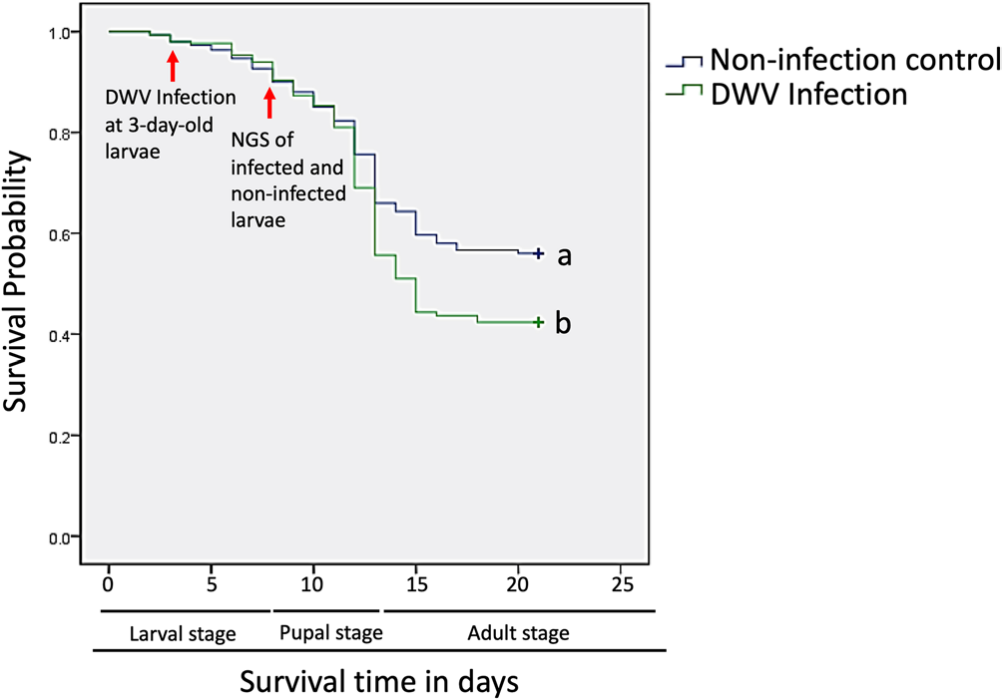
A survival curve was constructed based on Kaplan–Meier analysis using the data of the deformed wing virus (DWV) infection (1.55 × 10^10^ copies/per larva) and noninfection control groups over 22 days (100 larvae/per repeat and total 3 replicates). A significant difference between these groups was observed during all stages in this experiment. Different letters are significantly different (chi-square = 9.785; 1 d.f.; *P* = 0.002).

Honey bee larvae were infected with 1.55 × 10^10^ copies/per larva at 3 days old (0 d.p.i.). The number of surviving larvae from both the infected and noninfected groups was recorded daily from 0 to 22 days after infection and analysed using Kaplan–Meier survival analysis. A similar survival rate for the DWV-infected and noninfected groups was observed, but a significantly lower survival rate (chi-square = 9.785; 1 d.f.; *P* = 0.002) was identified in the DWV-infected group during the period between the late larval stage and prepupal stage (Fig. 1).

**Figure 2:**
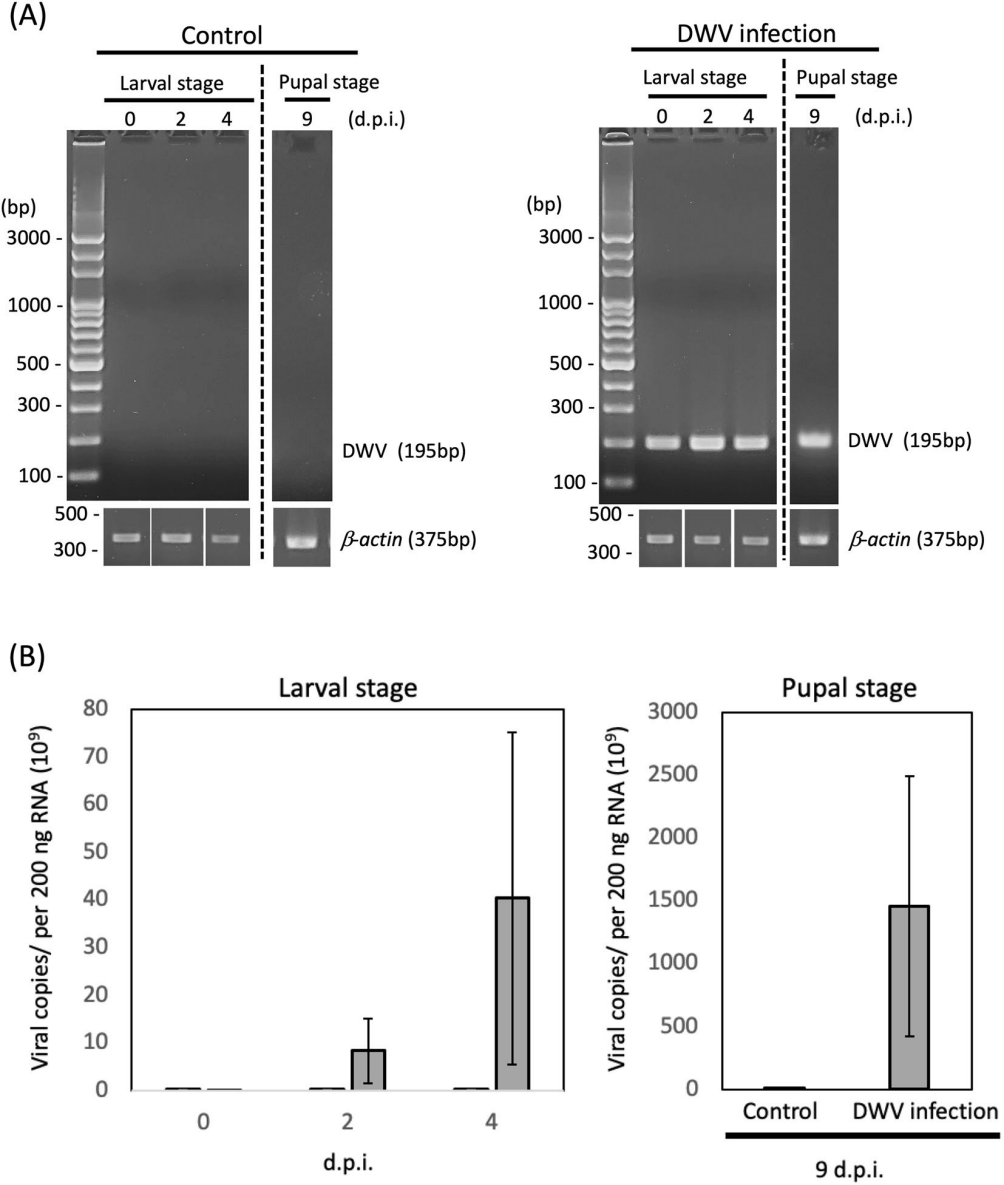
Detection of DWV infection by **(A)** RT-PCR and **(B)** quantitative RT-PCR (qRT-PCR) based on a plasmid standard curve. The samples were collected at 0, 2, 4 and 9 d.p.i. The copies of DWV were increased from 2 d.p.i. to 6 d.p.i. and continuously increased in the pupal stage. Full-length gels are presented in Supplementary Figs. 4 and 5.

The replication of DWV was detected in the infected group at the larval stage (0, 2 and 4 d.p.i.) and pupal stage (9 d.p.i.) (Fig. 2). The DWV copies gradually increased from 2 d.p.i. to 4 d.p.i. and dramatically increased to the pupal stage (9 d.p.i.). Based on the results, the DWV copies changed dramatically from the last phase of the larval stage (4 d.p.i.) to the pupal stage (9 d.p.i.), and the mortality was increased after the pupal stage (Figs. 1 and 2); therefore, the transcriptomic experiment focused on the last phase of the larval stage to investigate the gene expression profile in this period.

The survival rates for both groups were similar until 7 days of age (4 d.p.i.); however, in the 10-day-old samples (pupal stage), the survival rate was greatly decreased in the infected group, while DWV copies increased significantly during the pupal stage. Honey bee eggs were exposed to DWV predominantly by virus transovum^25^; thus, honey bee eggs show a high risk of DWV infection once larvae hatch. Ten days after DWV infection, we observed that the survival rate was substantially decreased in the infected group, suggesting that DWV may have negative effects starting in the early life stage of honey bees, revealing the risk of infection to the honey bee population (Fig. 1).

### Statistical analysis of next-generation sequencing

In total, 47,569,753 and 44,405,693 raw reads were generated from the DWV-infected and noninfected groups, respectively. Quality paired-end reads were obtained by adaptor trimming and quality checking. The quality reads were then mapped to the honey bee gene index and corresponding annotation database (GCF_000002195.4_Amel_4.5_genomic.gff). The average mapping rates to map RNA transcripts were 82.5% for the DWV-infected group and 91% for the noninfected group (Supplementary Table 3). The mapped reads were located in 9820 genes of honey bee mRNA and then were processed for further DEG analysis. The PE reads were further mapped to the genomes of common honey bee viruses and confirmed that the control and DWV infection groups did not have other honey bee viral infections (Supplementary Table 4).

### Detection of differentially expressed genes (DEGs)

**Figure 3:**
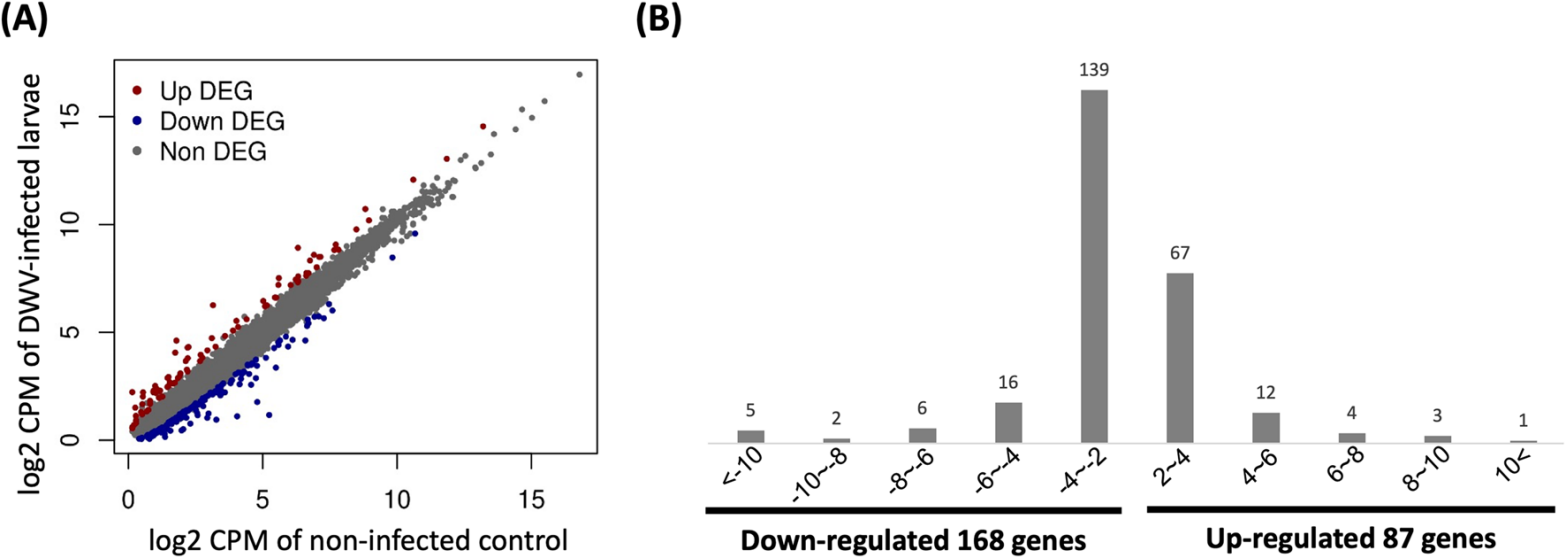
Overview of the fold change (log2 ratio ≥ 1) distribution of differentially expressed genes (DEGs). **(A)** Expression levels of the contigs in noninfected and DWV-infected larvae. **(B)** Analysis of DEGs identified from noninfected and DWV-infected samples. Downregulated genes are shown on the left side, and upregulated genes are shown on the right side. The expressed contigs with fold change ≥ 2 or ≤ -2 are listed, and the contig numbers with corresponding fold change are shown above each bar.

To identify the significantly differentially expressed genes, expression profile comparisons between the infected and noninfected groups were performed. The DEG analysis results showed that most of the gene expression levels were similar between these groups; however, some of the genes showed different expression levels (Fig. 3; Supplementary Fig. 6). Two hundred sixty-five genes (0.26%) exhibited significant fold changes in the expression profile (fold change ≥ 2 or ≤ -2) between the DWV-infected and noninfected groups (Fig. 3A,B; Supplementary Tables 5 and 6). Among these DEGs, 168 downregulated and 87 upregulated genes were identified (Fig. 3B; Supplementary Tables 5 and 6). The fold changes of most of the down- and upregulated genes (67 genes and 139 genes, respectively) ranged from -2 to -4 and 2 to 4 (Fig. 3B; Supplementary Tables 5 and 6). The DEGs with higher fold changes (fc ≥ 4 or ≤ -4) were summarized; of these DEGs, 22.99% (20 genes) of genes were more highly upregulated, and 17.26% (29 genes) were more highly downregulated (Tables 1 and 2). Thus, the transcriptomes of DWV-infected and noninfected honey bee larvae are moderate, but some physical mechanisms may be subjected to the effect of DWV infection.

**Table 1:** List of upregulated genes (fold change ≥ 4) identified in the DWV-infected honey bee larvae library.

No	Gene symbol	Location	Annotation	Gene type	Fold change	KO annotation
1	LOC102656630	NC_037639.1 (10986767..10989283)	Uncharacterized LOC102656630	ncRNA	34.50	
2	LOC725305	NC_037638.1 (27575255..27577314)	Uncharacterized LOC725305	Protein coding	9.59	
3	LOC102656570	NC_037644.1 (9765896..9768671)	Glucose dehydrogenase [FAD, quinone]	Protein coding	9.59	
4	LOC100577585	NC_037638.1 (6125693..6132159, complement)	Uncharacterized LOC100577585	Protein coding	9.39	
5	LOC100577331	NC_037647.1 (5365831..5367042)	Cell wall integrity and stress response component 1	Protein coding	7.90	
6	LOC113218981	NC_037646.1 (6584793..6586590, complement)	E3 ubiquitin-protein ligase MARCH8-like	Pseudo	6.70	
7	LOC724464	NC_037651.1 (1453144..1459475)	Cuticular protein	Protein coding	6.67	
8	LOC552024	NC_037645.1 (8626452..8629999)	Myo-inositol 2-dehydrogenase	Protein coding	6.18	K00010
9	LOC113219275	NC_037638.1 (12578526..12578968, complement)	Uncharacterized LOC113219275	ncRNA	5.43	
10	LOC551782	NC_037642.1 (598474..602169, complement)	Bestrophin-4	Protein coding	5.17	K22204
11	LOC413924	NC_037647.1 (10700617..10702820, complement)	Glyceraldehyde-3-phosphate dehydrogenase	Protein coding	5.11	K00134
12	LOC107965534	NC_037651.1 (5066923..5068437)	Protein Fer3-like	Protein coding	4.99	K22400
13	LOC113218572	NC_037647.1 (10291785..10293155)	Uncharacterized LOC113218572	Protein coding	4.87	
14	LOC102655275	NC_037638.1 (11942593..11953074, complement)	Uncharacterized LOC102655275	ncRNA	4.70	
15	LOC102656842	NC_037643.1 (13368509..13370515, complement)	Uncharacterized LOC102656842	ncRNA	4.40	
16	LOC725804	NC_037645.1 (10430848..10433690, complement)	Cuticle protein 18.7	Protein coding	4.24	
17	LOC100576935	NC_037646.1 (8198101..8205488, complement)	Uncharacterized LOC100576935	Protein coding	4.05	
18	LOC102656669	NC_037638.1 (12577483..12580393)	Ctenidin-1-like	Protein coding	4.04	
19	LOC113219409	NC_037638.1 (10363934..10369500)	Uncharacterized LOC113219409	ncRNA	4.03	
20	LOC100578187	NC_037638.1 (1968930..1971969, complement)	Uncharacterized LOC100578187	ncRNA	4.02

**Table 2:** List of downregulated genes (fold change ≤ − 4) identified in the DWV-infected honey bee larvae library.

No	Gene symbol	Location	Annotation	Gene type	Fold change	KO annotation
1	LOC107963975	NC_037638.1 (13283980..13285235)	Uncharacterized LOC107963975	Protein coding	−29.55	
2	LOC102655555	NC_037647.1 (12296588..12305191, complement)	Uncharacterized LOC102655555	ncRNA	−15.22	
3	LOC413908	NC_037650.1 (10728080..10731750, complement)	Cytochrome P450 6A1	Protein coding	−13.29	K14999
4	LOC100576697	NC_037638.1 (23954915..23963214)	Uncharacterized LOC100576697	ncRNA	−13.19	
5	LOC724565	NC_037643.1 (3145515..3148360, complement)	Trypsin-7	Protein coding	−10.97	
6	LOC409751	NC_037651.1 (6389782..6392462)	Multiple inositol polyphosphate phosphatase 1	Protein coding	−9.14	K03103
7	LOC100577819	NC_037643.1 (5585899..5588396, complement)	Uncharacterized LOC100577819	Protein coding	−8.59	
8	LOC102656882	NC_037651.1 (5937498..5939234, complement)	Cytochrome P450 9e2-like	Protein coding	−7.91	K15003
9	Est-6	NC_037640.1 (9461728..9464050)	Venom carboxylesterase-6	Protein coding	−7.30	K12298
10	LOC113218894	NC_037644.1 (10166236..10175388, complement)	Uncharacterized LOC113218894	ncRNA	−7.27	
11	LOC102655781	NC_037653.1 (4736319..4743867, complement)	Uncharacterized LOC102655781	ncRNA	−7.05	
12	LOC410850	NC_037639.1 (12443562..12446826)	Coiled-coil domain-containing protein 63-like	Protein coding	−6.75	K23732
13	LOC410317	NC_037648.1 (11450640..11678693, complement)	Small conductance calcium-activated potassium channel protein	Protein coding	−6.26	K04944
14	LOC725087	NC_037650.1 (10762729..10766334, complement)	Probable cytochrome P450 6a14	Protein coding	−5.82	K14999
15	LOC410405	NC_037650.1 (2519228..2524033)	Cytochrome P450 18a1	Protein coding	−5.62	K14985
16	LOC102656658	NC_037644.1 (3642775..3659414, complement)	Uncharacterized LOC102656658	ncRNA	−5.57	
17	LOC410406	NC_037650.1 (2532325..2536231)	Zinc metalloproteinase nas-13	Protein coding	−5.50	
18	LOC100576130	NC_037647.1 (11434939..11439457, complement)	Diuretic hormone 44	Protein coding	−5.26	
19	LOC724900	NC_037640.1 (12753760..12765869)	Uncharacterized LOC724900	Protein coding	−5.01	
20	LOC102654839	NC_037651.1 (7556583..7571148)	Uncharacterized LOC102654839	Protein coding	−4.97	
21	LOC726459	NC_037652.1 (7781687..7783890, complement)	Odorant receptor 13a	Protein coding	−4.94	
22	crh-BP	NC_037645.1 (2187253..2195465, complement)	Corticotropin releasing hormone binding protein	Protein coding	−4.77	
23	LOC727193	NC_037648.1 (8288010..8291476, complement)	Lipase member H-A	Protein coding	−4.73	
24	LOC113219002	NC_037646.1 (2711025..2713806)	Uncharacterized LOC113219002	Protein coding	−4.63	
25	LOC551044	NC_037642.1 (11669303..11678572)	Glucose dehydrogenase [FAD, quinone]	Protein coding	−4.58	
26	LOC411894	NC_037645.1 (12183077..12209596, complement)	Dynein beta chain, ciliary	Protein coding	−4.47	K10408
27	LOC413346	NC_037638.1 (5691449..5701789, complement)	Endoglucanase E-4	Protein coding	−4.06	K01179
28	LOC100577043	NC_037638.1 (23246496..23248301)	Uncharacterized LOC100577043	Protein coding	−4.02	
29	LOC725264	NC_037646.1 (4169782..4364474, complement)	Uncharacterized LOC725264	Protein coding	−4.00	

### Gene ontology (GO) analysis of differentially expressed genes

**Figure 4:**
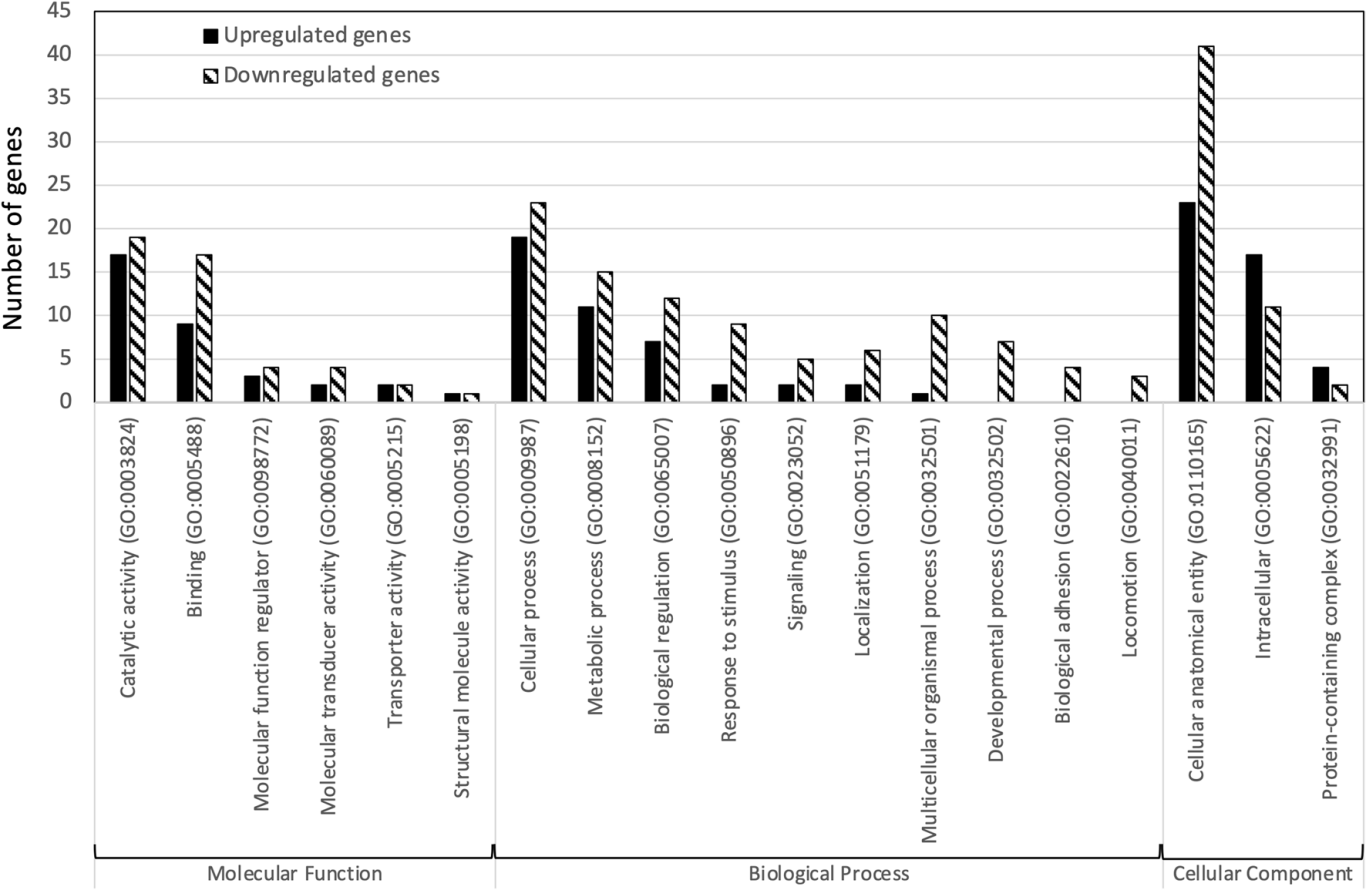
Gene ontology (GO) analysis of differentially expressed genes (DEGs) from DWV-infected honey bee larvae with a fold change (fc) ≥ 2. In total, 909 DEGs were subjected to GO analysis, and 564 DEGs (65.1%) were categorized into three groups—molecular function, cellular component and biological process; however, 318 DEGs (34.9%) were unannotated in the GO database.

To detect the effects of DWV-infected honey bee larvae, 208 DEGs were subjected to gene ontology (GO) analysis. One hundred forty-one DEGs (55.3%) were categorized into three groups: molecular function, cell component and biological process (Fig. 4; Supplementary Tables 7 and 8). The black bar indicates upregulated genes, and the white bar indicates downregulated genes (Fig. 4). For upregulated genes, the cellular anatomical entity (GO:0110165) in the cellular component category was the group with the most abundant DEGs, followed by cellular process (GO:0009987) in the biological process category and catalytic activity (GO:0003824) in the molecular function category and intracellular (GO:0005622) in the cellular component category (Fig. 4; Supplementary Table 7). For downregulated genes, the cellular anatomical entity (GO:0110165) in the cellular component category was also the group with the most abundant DEGs, followed by cell process (GO:0009987) in the biological process category and catalytic activity (GO:0003824) in the molecular function category (Fig. 4; Supplementary Table 8).

Based on the GO enrichment analysis results, the amino acid binding (GO:0016597), organic acid binding (GO:0043177) and carboxylic acid binding (GO:0031406) in the molecular function category were significantly upregulated with fold enrichments of 33.2, 25.9 and 25.9, respectively (FDR < 0.05; Table 3), while the external side of plasma membrane (GO:0009897) and basement membrane (GO:0005604) in the cellular component category, cell–cell adhesion via plasma-membrane adhesion molecules (GO:0098742) in the biological process category and cell surface (GO:0009986) in the cellular component category were downregulated with highly fold enrichments of 28.5, 21.7, 12.7 and 10.8, respectively (FDR < 0.05; Table 4). The GO enrichment results indicated that DWV infection may influence the cellular component and membrane structure and affect the binding affinity of immaterial or material entities with granularity of cells. Therefore, impacting the transmission of the signal from one side of the cell membrane to the other side initiates a change in cell activity. Rabies virus (RABV) infectivity is drastically decreased after metabotropic glutamate receptor subtype 2 (mGluR2) siRNA knockdown in cells, and mGluR2 modulates a functional cellular entry receptor for RABV^48^. GO analysis revealed a negative effect of the plasma membrane and cell surface, which may suppress DWV infection. Additionally, the catalytic activity and response to stimulus-related pathways were enriched, suggesting that honey bee larvae attempted to respond to DWV infection. Based on the survival data, honey bee larvae successfully escaped DWV infection; however, the protective mechanism may fail to work and lead to a lower survival rate during metamorphosis (Fig. 1).

**Table 3:** PANTHER gene ontology (GO) enrichment analysis of upregulated genes.

Annotation data set^a^	GO term	No of genes	Fold enrichment	FDR^¶^
Molecular function	Amino acid binding (GO:0016597)	3	33.15	7.14E−02
	Organic acid binding (GO:0043177)	3	25.9	7.01E−02
	Carboxylic acid binding (GO:0031406)	3	25.9	4.67E−02

Displaying only results for FDR p < 0.05.

^¶^p-value from Fisher's exact test with false discovery rate (FDR) correction for multiple testing.

^a^PANTHER GO-Slim analysis.

**Table 4:** PANTHER gene ontology (GO) enrichment analysis of downregulated genes.

Annotation data set^a^	GO term	No of genes	Fold enrichment	FDR^¶^
Molecular function	Calcium ion binding (GO:0005509)	9	6.76	2.76E−02
	Tetrapyrrole binding (GO:0046906)	7	7.59	4.73E−02
	Heme binding (GO:0020037)	7	7.64	6.78E−02
Biological process	Cell adhesion (GO:0098602)	9	7.81	2.47E−02
	Biological adhesion (GO:0022610)	9	7.46	1.75E−02
	Cell–cell adhesion (GO:0098609)	7	9.84	2.62E−02
	Cell–cell adhesion via plasma-membrane adhesion molecules (GO:0098742)	6	12.65	2.29E−02
Cellular component	Cell periphery (GO:0071944)	31	2.61	3.53E−04
	Intrinsic component of membrane (GO:0031224)	22	2.27	1.99E−02
	Plasma membrane (GO:0005886)	22	2.36	1.63E−02
	Intracellular anatomical structure (GO:0005622)	21	0.49	1.81E−03
	Extracellular region (GO:0005576)	21	3.06	2.46E−03
	Integral component of membrane (GO:0016021)	21	2.21	3.29E−02
	Organelle (GO:0043226)	17	0.47	6.25E−03
	Intracellular organelle (GO:0043229)	14	0.41	1.50E−03
	Membrane-bounded organelle (GO:0043227)	14	0.44	8.08E−03
	Intrinsic component of plasma membrane (GO:0031226)	13	3.79	6.89E−03
	Integral component of plasma membrane (GO:0005887)	12	3.6	1.51E−02
	Intracellular membrane-bounded organelle (GO:0043231)	11	0.36	1.85E−03
	External encapsulating structure (GO:0030312)	9	4.45	2.10E−02
	Extracellular matrix (GO:0031012)	9	5	1.25E−02
	Cell surface (GO:0009986)	5	10.84	1.57E−02
	Nucleus (GO:0005634)	4	0.22	7.05E−03
	Collagen-containing extracellular matrix (GO:0062023)	4	14.12	1.94E−02
	Basement membrane (GO:0005604)	3	21.68	3.49E−02
	External side of plasma membrane (GO:0009897)	3	28.46	2.03E−02

Displaying only results for FDR P < 0.05.

^¶^p-value from Fisher's exact test with false discovery rate (FDR) correction for multiple testing.

^a^PANTHER GO-Slim analysis.

### KEGG analysis of differentially expressed genes

Pathway analysis was applied using the KEGG (Kyoto Encyclopedia of Genes and Genomes) tool to detect the effect of DWV infection on the gene expression of larvae. One hundred eleven (38 upregulated and 73 downregulated genes) of 255 DEGs (43.53%) were annotated by KEGG pathway mapping (Supplementary Fig. 7; Supplementary Tables 9 and 10). Among these mapped DEGs, pathways of many metabolism-related enzymes possessed the highest numbers of hits, followed by “biosynthesis of secondary metabolites”, “glycine, serine and threonine metabolism”, “Rap1 signaling pathway”, “cAMP signaling pathway”, “calcium signaling pathway” and “inositol phosphate metabolism”.

Further analysis of the DEGs involved in these pathways revealed two upregulated DEGs [myo-inositol 2-dehydrogenase (*iolG*, LOC552024) and glyceraldehyde-3-phosphate dehydrogenase (*GAPDH*, LOC413924)] were involved in 5 and 12 pathways, respectively (Table 1; Supplementary Table 9). From our data, *IolG* was mapped to 5 pathways, including microbial metabolism in diverse environments (ko01120) and inositol phosphate metabolism (ko00562). The *iolG* gene is involved in inositol catabolism in *Aerobacter aerogenes*^49^. Inositol monophosphatase mediates the synthesis of myo-inositol, which is likely involved in lipid metabolism^50^. A recent study also demonstrated that inositol phosphates enhances RNA virus (HIV-1) assembly and prevents viruses from cellular defence^51^, suggesting that upregulation of *IolG* after honey bee infection with DWV may facilitate viral assembly and spread. According to the analysis, *GAPDH* is involved in 12 different pathways, such as microbial metabolism in diverse environments (ko01120), carbon metabolism (ko01200), and glycolysis/gluconeogenesis (ko00010), indicating the importance of this gene in the biological function of the cells^52^. *GAPDH* is a suitable housekeeping gene in honey bees; however, *GAPDH* is associated with and regulated after viral infection^53,54^. Therefore, hijacking host cellular resources by upregulating *IolG* and *GAPDH* after honey bee infection with DWV may facilitate viral infection.

Regarding the downregulated DEGs, multiple inositol polyphosphate phosphatase 1 (*MINPP1*, LOC409751) involved in 3 pathways (Table 2; Supplementary Table 10) was more relevant to viral infection. *MINPP1* is an enzyme that hydrolyses abundant metabolites, such as inositol pentakisphosphate and inositol hexakisphosphate^55^. Inositol hexakisphosphate stimulates both immature and mature HIV-1 particle assembly to become an infectious form^51^. Suppressing *MINPP1* expression may be a mechanism of honey bees against DWV infection.

### qRT-PCR validation of DEGs

**Figure 5:**
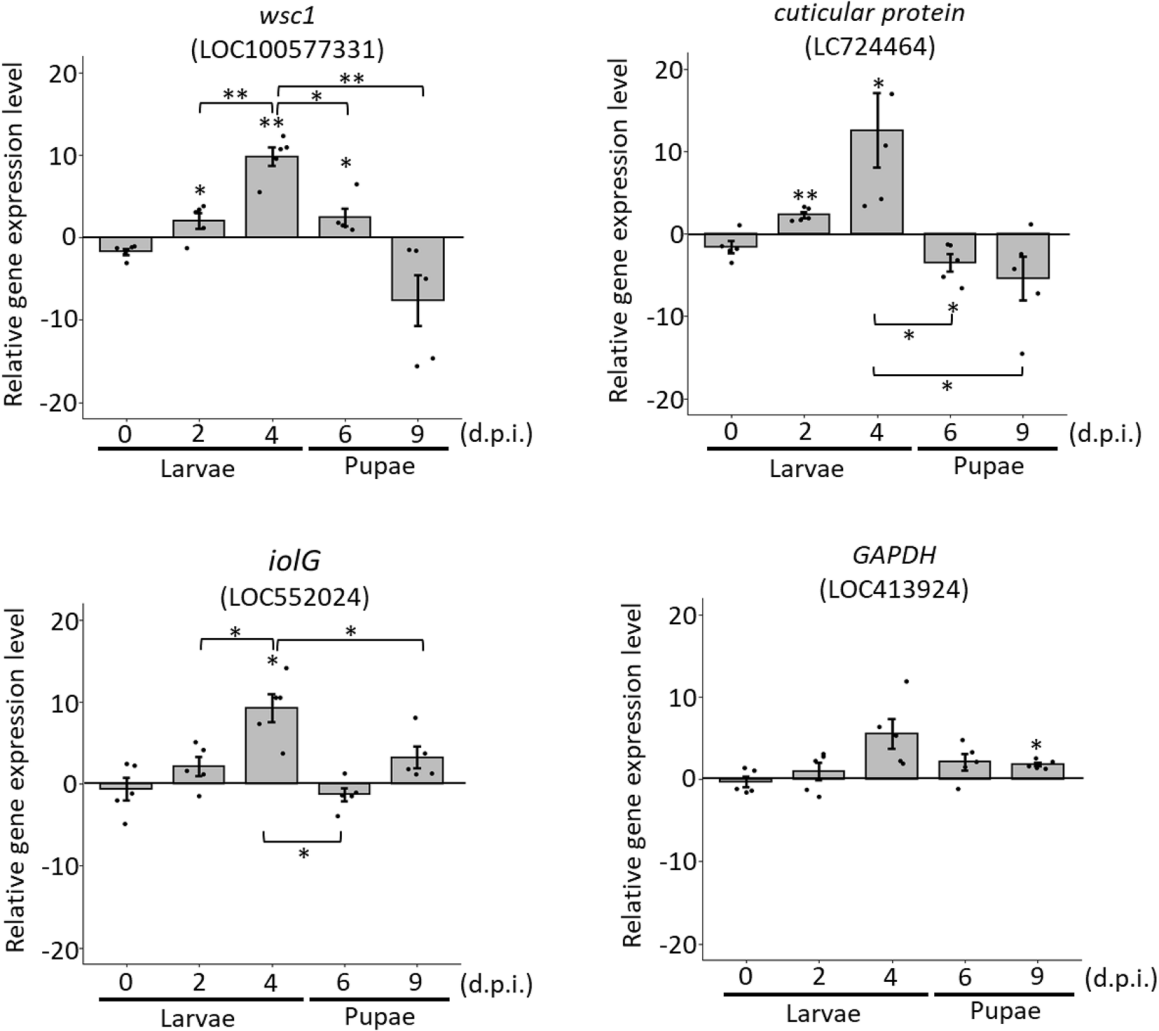
Validation of upregulated genes at 0, 2, 4, 6 and 9 d.p.i. The mean ± SD was calculated for relative gene expression levels using the 2^−ΔΔCt^ method (Livak &Schmittgen 2001). All experiments were performed in five replicates. *P < 0.05; **P < 0.01.

**Figure 6:**
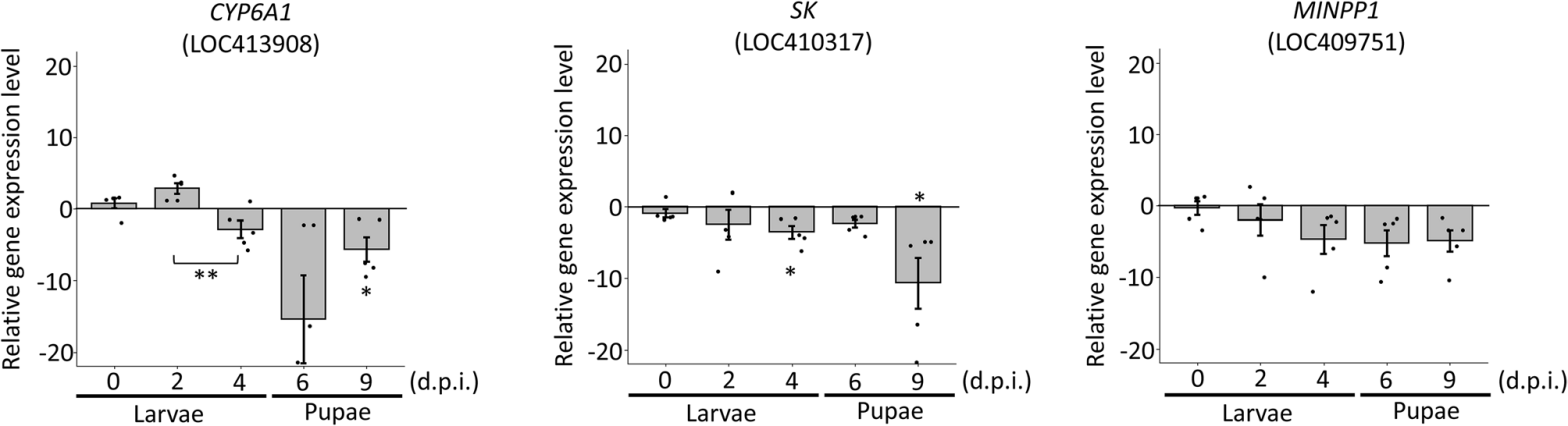
Validation of downregulated genes at 0, 2, 4, 6 and 9 d.p.i. The mean ± SD was calculated for relative gene expression levels using the 2^−ΔΔCt^ method (Livak &Schmittgen 2001). All experiments were performed in five replicates. *P < 0.05; **P < 0.01.

In total, 20 genes were upregulated and 29 genes were downregulated in DWV-infected honey bee larvae. To further validate the transcriptome data, four upregulated [*wsc1* (LOC100577331), *cuticular protein* (LOC724464), *iolG* (LOC552024) and *GAPDH* (LOC413924)] and three downregulated genes [*CYP6A1* (LOC413908), *SK* (LOC410317) and *MINPP1* (LOC409751)] were subjected to RT-qPCR validation at different d.p.i. As expected, the related gene expression levels of all the selected DEGs showed similar predictions (Figs. 5 and 6). Regarding the upregulated DEGs, four genes increased the expression levels from 0 to 4 d.p.i. and reached a high peak at 4 d.p.i., while a declining pattern was observed at the pupal stage (Fig. 5). Most of the downregulated genes were consistent with expectations; the related gene expression levels were suppressed from 0 to 4 d.p.i., while *CYP6A1* altered the related expression level from upregulation (2 d.p.i.) to downregulation (after 4 d.p.i.) (Fig. 6).

Proteins containing WSC domains have many biological functions, such as mediating intracellular responses to environmental stress^56,57^. Interestingly, the functions of *wsc*-related genes play a role in the heat shock response involved in the Pkc1-MPK1 signalling pathway^57^. In honey bees, the MAPK cascade transmits signals from the outer cell surface to the nucleus is an antiviral mechanism related to endocytosis^58^. The *wsc1* gene was upregulated from 2 to 6 d.p.i. and then was suppressed at 9 d.p.i. (fc = − 7.61), indicating that DWV infection triggers the host defence system; however, downregulation of the *wsc1* gene reflects a significant drop in the survival rate of DWV-infected honey bees in the pupal stage.

Cuticular protein is the protein plays an important role in the protection of insects. During DWV infection, the *cuticular protein* gene of honey bees was upregulated at 2 d.p.i. to 4 d.p.i. (fc = 2.37 and 12.63, respectively) but downregulated at 6 d.p.i. to 9 d.p.i. (fc = − 3.46 and − 5.42, respectively) (Fig. 5). Additionally, *cuticular protein* is downregulated when honey bees are infected with the microsporidian *Nosema ceranae*^59^, and the data are similar to the related expression level of *cuticular protein* in honey bee pupae infected with DWV, suggesting a similar outcome under this gene suppression. As mentioned above, inositol phosphates enhances RNA virus (HIV-1) assembly and prevents viruses from cellular defence^51^. Additionally, *GAPDH* is associated with and regulated after viral infection^53,54^. Furthermore, relative quantitative RT-PCR showed that the *iolG* gene was upregulated at 2 d.p.i., 4 d.p.i. and 9 d.p.i. (fc = 2.15, 9.29 and 3.26, respectively), and *GAPDH* was upregulated at 4 to 6 d.p.i (fc = 5.62 and 2.15, respectively). Therefore, upregulation of *IolG* and *GAPDH* after honey bee infection with DWV may facilitate the viral infection process.

Regarding the three downregulated genes, the related expression level showed a continuous declining pattern from the beginning (larval stage) of DWV infection to the pupal stage, except that *CYP6A1* was upregulated at 2 d.p.i. (fc = 2.89) (Fig. 6). *CYP6A1* is a member of the cytochrome P450 family and performs various enzyme reactions. Previous studies have indicated that *Drosophila melanogaster* P450 enzymes are related to developmental processes and are involved in the detoxification of foreign compounds^60^. In honey bees, *CYP6A1* upregulation may be induced by DWV infection; however, downregulation is mostly observed during DWV infection, suggesting that insufficient detoxifying proteins, such as *CYP6A1*, may reduce the survival fitness of honey bees.

The SK channel plays a fundamental role in all excitable cells, is potassium selective and is activated by increased intracellular calcium levels, such as during action potentials^61^. Additionally, dendritic cells (DCs), which are controlled by Ca^2+^ signalling, play an important role in innate and adaptive immunity. Therefore, alterations of cytosolic Ca^2+^ can trigger immune suppression or switch off DC activity^62^. From our data, *SK* was downregulated at all time points, and significant downregulation was observed at 4 d.p.i. and 9 d.p.i. (fc = − 3.51 and − 10.64, respectively), indicating that the signals for the immune system may be dysregulate after DWV infection.

As mentioned above, *MINPP1* hydrolyses inositol pentakisphosphate and inositol hexakisphosphate^55^. Inositol hexakisphosphate stimulates both immature and mature HIV-1 particle assembly to become an infectious form^51^. Based on the results of relative quantitative RT-PCR, *MINPP1* expression was downregulated at all time points. Suppressed *MINPP1* expression may be a mechanism of honey bees against DWV infection. Therefore, the gene expression pattern shows that continuous downregulation of *MINPP1* in the pupal stage may affect the fitness of honey bees during pathogen infections.

## Conclusions

In this study, the effect of DWV on honey bee larvae was evaluated based on transcriptomic level assessment. DEG identification showed that the transcriptomes of DWV-infected and noninfected honey bee larvae were moderate, while some physical mechanisms may be subjected to the effect of DWV infection. By validating these DEGs, *wsc1*, *cuticular protein* and *iolG* were confirmed to be significantly upregulated, and *SK* was significantly downregulated at 4 d.p.i. and continuously to the pupal stage. Additionally, these DEGs were significantly regulated in the pupal stage, indicating the potential effects of the gene expression levels from the larval to the pupal stages. In conclusion, DWV infection in the honey bee larval stage may influence the gene expression levels from larvae to pupae and reduce the survival rate of the pupal stage. This information highlights the consequences of DWV prevalence in honey bee larvae for apiculture. Therefore, detection and management strategies for DWV-infected honey bee colonies at an early stage must be developed to avoid colony losses.

## Supplementary Information

Supplementary Information.

## Data Availability

The datasets generated in this study are available in the Supplementary Information files (available online). The sequence data were submitted to the NCBI Sequence Read Archive (BioProject accession: PRJNA669279) or are available from the corresponding author (Dr. Yu-Shin Nai) by request.
