# Supplementary material for: Transcriptome-level assessment of the impact of deformed wing virus on honey bee larvae
Source: Sci Rep. 2021 Jul 22;11:15028. doi: 10.1038/s41598-021-94641-3 (PMC8298419; doi:10.1038/s41598-021-94641-3)
Supplement: Supplementary file 1 — Supplementary Information. [file 41598_2021_94641_MOESM1_ESM.docx]

**Transcriptome-level assessment of the impact of deformed wing virus on honey bee larvae**

Zih-Ting Chang^1¶^, Yu-Feng Huang^2¶^, Yue-Wen Chen^1¶^, Ming-Ren Yen^3^, Po-Ya Hsu^1^, Tzu-Han Chen^3^, Yi-Hsuan Li^3^, Kuo-Ping Chiu^2^, and Yu-Shin Nai^3*^

^1.^ Department of Biotechnology and Animal Science, National Ilan University, Yilan City, 260, Taiwan

^2.^ Genomics Research Center, Academia Sinica, Taipei City, 115, Taiwan

^3.^ Department of Entomology, National Chung Hsing University, Taichung City, 402, Taiwan

^¶^ These authors contributed equally to this work.

* Corresponding author

**Correspondence to:**

**Yu-Shin Nai, Ph.D.**

Department of Entomology, National Chung Hsing University, Taichung City, 402, Taiwan

Tel: +886-4-2284-0361 ext 513

E-mail: [ysnai@nchu.edu.tw](mailto:ysnai@nchu.edu.tw)

**Supplementary Table 1.** Seven honey bee viral gene-specific primer sets

| **Target** | **Primer** | **Sequence (5' to 3')** | **Amplicon Length (bp)** | **Reference** |
| --- | --- | --- | --- | --- |
| Acute Bee Paralysis Virus (ABPV) | ABPV1 | TTATGTGTCCAGAGACTGTATCCA | 900 | ^1^ |
|  | ABPV2 | GCTCCTATTGCTCGGTTTTTCGGT |  |  |
| Black Queen Cell Virus (BQCV) | BQCV1 | TGGTCAGCTCCCACTACCTTAAAC | 700 | ^1^ |
|  | BQCV2 | GCAACAAGAAGAAACGTAAACCAC |  |  |
| Chronic Bee Paralysis Virus (CBPV) | CBPV1 | AGTTGTCATGGTTAACAGAGTACGAG | 455 | ^2^ |
|  | CBPV2 | TCTAATCTTAGCACGAAAGCCGAG |  |  |
| Deformed Wing Virus (DWV) | DWV-F | CTTACTCTGCCGTCGCCCA | 194 | ^3^ |
|  | DWV-R | CCGTTAGGAACTCATTATCGCG |  |  |
| Israel Acute Paralysis Virus (IAPV) | IAPV-F | AGACACCAATCACGGACCTCAC | 475 | ^4^ |
|  | IAPV-R | AGATTTGTCTGTCTCCCAGTGCACA |  |  |
| Sacbrood Virus (SBV) | SBV-F | GCTGAGGTAGGATCTTTGCGT | 824 | ^3^ |
|  | SBV-R | TCATCATCTTCACCATCCGA |  |  |
| Varroa destructor Virus-1 (VDV-1) | VDV-F_RTPCR_ | CGAAACGAAGAGAGCATGTAT | 1129 | ^5^ |
|  | VDV-R_RTPCR_ | CGACTCTTCCCCAGCTAAG |  |  |
| *Apis mellifera* actin (β-actin) | actin-F | CCCGAGGCTCTTTTCCAACC | 199 | This study |
|  | actin-R | TGGATGGTGCTAGGGCAGTG |  |  |
| *Apis mellifera* actin (18S) | Hb-18S-F | GTAACCCGTTGAACCCCATT | 151 | This study |
|  | Hb-18S-R | CCATCCAATCGGTAGTAGCG |  |  |

| **Supplementary Table 2.** Primer list to screen for gene expression validation | | | | | |
| --- | --- | --- | --- | --- | --- |
| **No.** | **Gene ID** | **Gene name** | **Primer name** | **Sequence (5' to 3')** | **Reference** |
| 1 | LOC100577331 | *wcs1* | LOC100577331-F | GCAGCGGCACACACTTCA | This study |
|  |  |  | LOC100577331-R | AGAATCTTCCGTTGGACAATCAA |  |
| 2 | LOC724464 | *cuticular protein* | LOC724464-F | TCGCAACGCTCAAGACAGAT | This study |
|  |  |  | LOC724464-R | AGTGAGAGGGTCTGCGTGTAAAA |  |
| 3 | LOC552024 | *iolG* | LOC552024-F | TCGAGGTATTTGGCCCTAAGG | This study |
|  |  |  | LOC552024-R | TGACTGGTAACACAGTGCATTGG |  |
| 4 | LOC413924 | *GAPDH* | LOC413924-F | CGGTGGGAAGAGGGTGATTA | This study |
|  |  |  | LOC413924-R | AGCATGATTCACCCCGTAAACT |  |
| 5 | LOC413908 | *CYP6A1* | LOC413908-F | CGTAGAATTGACCGATGCTTTCC | This study |
|  |  |  | LOC413908-R | AACCAGCGGCGAAGAAAAC |  |
| 6 | LOC410317 | *SK* | LOC410317-F | TGTGAAATGTTCCACCACTGTTT | This study |
|  |  |  | LOC410317-R | ACCTATGACCTTTGAGCACTTCCT |  |
| 7 | LOC409751 | *MINPP1* | LOC409751-F | GCTATGACGGCGACCTTATATTAC | This study |
|  |  |  | LOC409751-R | CCGTGTATGACCGTATATCGTTC |  |

**Supplementary Table 3.** Mappability of the RNA-seq data to the honey bee genome

| **Sample name** | **Raw reads** | **Trimmed reads** | **Mapped reads** | **Mappability** |
| --- | --- | --- | --- | --- |
| Control-1 | 20,464,054 | 20,099,462 | 18,389,002 | 91% |
| Control-2 | 23,941,639 | 23,618,321 | 21,462,098 | 91% |
| DWV-1 | 25,799,203 | 25,479,883 | 20,782,713 | 82% |
| DWV-2 | 21,770,550 | 21,463,820 | 17,785,754 | 83% |

**Supplementary Table 4.** Screening of transcriptomic data for honey bee larvae by mapping to the genomes of 7 honey bee-infecting viruses

| **Sample name** | **No. of Trimmed reads** | **%DWV** | **%VDV** | **%BQCV** | **%KBV** | **%IAPV** | **%ABPV** | **%SBV** |
| --- | --- | --- | --- | --- | --- | --- | --- | --- |
| Control-1 | 20099462 | 0.0% (5) | - | 0% (13) | - | - | - | - |
| Control-2 | 23618321 | - | - | 0% (27) | - | - | - | - |
| DWV infection -1 | 25479883 | 7% (1792163) | 0% (280) | 0% (11) | - | - | - | - |
| DWV infection -2 | 21463820 | 5.6% (1199748) | 0% (159) | 0% (12) | - | - | - | - |

-= unmapped

Parentheses= mapped reads

**Supplementary Table 5**. List of upregulated genes identified in the DWV-infected honey bee larvae library

| **Gene symbol** | **Gene ID** | **CPM**  **control1** | **CPM**  **control2** | **CPM**  **infected_1** | **CPM**  **infected_2** | **logCPM** | **logFC** | **Fold change** | **PValue** | **FDR** |
| --- | --- | --- | --- | --- | --- | --- | --- | --- | --- | --- |
| LOC102656630 | 102656630 | 0.13 | 0.07 | 1.28 | 6.10 | 0.8973 | 5.1084 | 34.49791 | 3.71E-10 | 2.15E-07 |
| LOC725305 | 725305 | 2.39 | 2.51 | 4.76 | 42.31 | 3.6915 | 3.2616 | 9.5905504 | 2.43E-06 | 0.0003842 |
| LOC102656570 | 102656570 | 9.28 | 6.46 | 29.81 | 121.21 | 5.3795 | 3.2616 | 9.5904877 | 2.71E-11 | 2.75E-08 |
| LOC100577585 | 100577585 | 0.19 | 0.20 | 0.31 | 3.38 | 0.006 | 3.2314 | 9.3916773 | 0.000565 | 0.0240787 |
| LOC100577331 | 100577331 | 0.38 | 0.54 | 0.73 | 6.59 | 1.0179 | 2.9815 | 7.897992 | 0.000252 | 0.0134975 |
| LOC113218981 | 113218981 | 0.54 | 0.37 | 1.18 | 4.95 | 0.801 | 2.7452 | 6.7046851 | 3.69E-05 | 0.0032924 |
| LOC724464 | 724464 | 2.80 | 1.89 | 7.80 | 23.53 | 3.1649 | 2.7383 | 6.672702 | 3.77E-09 | 1.42E-06 |
| LOC552024 | 552024 | 83.85 | 72.87 | 291.17 | 677.09 | 8.1355 | 2.6271 | 6.1776578 | 1.60E-14 | 3.14E-11 |
| LOC113219275 | 113219275 | 0.08 | 0.34 | 1.13 | 1.25 | -0.4437 | 2.44 | 5.4263934 | 5.15E-05 | 0.0041484 |
| LOC551782 | 551782 | 3.69 | 3.51 | 34.86 | 2.33 | 3.484 | 2.3692 | 5.1665739 | 0.002219 | 0.0564566 |
| LOC413924 | 413924 | 4.55 | 2.87 | 21.23 | 16.65 | 3.5043 | 2.3542 | 5.1131612 | 5.86E-16 | 2.88E-12 |
| LOC107965534 | 107965534 | 0.32 | 0.54 | 1.78 | 2.59 | 0.4109 | 2.3184 | 4.9879091 | 1.30E-06 | 0.0002234 |
| LOC113218572 | 113218572 | 0.19 | 0.02 | 0.39 | 0.66 | -1.5008 | 2.2831 | 4.8672672 | 0.005091 | 0.0939644 |
| LOC102655275 | 102655275 | 0.62 | 1.38 | 2.15 | 7.31 | 1.5104 | 2.2338 | 4.7038603 | 0.000161 | 0.0097556 |
| LOC102656842 | 102656842 | 0.16 | 0.05 | 0.44 | 0.49 | -1.5962 | 2.1387 | 4.4035953 | 0.002754 | 0.0660731 |
| LOC725804 | 725804 | 0.24 | 1.57 | 3.82 | 3.93 | 1.276 | 2.0844 | 4.2410039 | 0.000945 | 0.032907 |
| LOC100576935 | 100576935 | 0.22 | 0.22 | 0.65 | 1.15 | -0.7567 | 2.017 | 4.04732 | 0.000807 | 0.0296822 |
| LOC102656669 | 102656669 | 0.73 | 1.38 | 3.35 | 5.21 | 1.4217 | 2.0146 | 4.0407784 | 4.03E-06 | 0.0005653 |
| LOC113219409 | 113219409 | 0.11 | 0.20 | 0.60 | 0.66 | -1.204 | 2.0092 | 4.0256825 | 0.001781 | 0.0501257 |
| LOC100578187 | 100578187 | 0.78 | 1.03 | 1.20 | 6.13 | 1.1834 | 2.0068 | 4.0188002 | 0.002068 | 0.054199 |
| LOC410520 | 410520 | 0.13 | 0.29 | 0.52 | 1.18 | -0.8252 | 1.9469 | 3.8554642 | 0.002978 | 0.0688582 |
| LOC724211 | 724211 | 57.68 | 37.38 | 64.31 | 300.17 | 6.8435 | 1.9388 | 3.83379 | 0.000128 | 0.0081371 |
| LOC726912 | 726912 | 0.38 | 0.39 | 1.70 | 1.25 | -0.0404 | 1.9326 | 3.8174474 | 7.45E-05 | 0.0054227 |
| Tpx-4 | Tpx-4 | 498.79 | 400.91 | 1133.66 | 2240.29 | 10.061 | 1.9069 | 3.7500591 | 4.58E-10 | 2.50E-07 |
| TpnCIIa | TpnCIIa | 2.31 | 1.18 | 5.42 | 7.37 | 2.0293 | 1.8733 | 3.663822 | 2.60E-06 | 0.000397 |
| LOC100578829 | 100578829 | 1.56 | 2.06 | 3.51 | 9.73 | 2.0728 | 1.863 | 3.6375631 | 8.32E-05 | 0.0058525 |
| LOC411665 | 411665 | 2.93 | 4.30 | 4.42 | 21.47 | 3.0443 | 1.8369 | 3.572504 | 0.000912 | 0.032228 |
| LOC102654968 | 102654968 | 1.08 | 0.96 | 1.47 | 5.70 | 1.197 | 1.8091 | 3.5042867 | 0.001929 | 0.052323 |
| LOC410149 | 410149 | 4.09 | 2.68 | 9.08 | 14.39 | 2.9187 | 1.7935 | 3.4665703 | 1.62E-07 | 3.62E-05 |
| LOC102653897 | 102653897 | 1.10 | 0.39 | 1.65 | 3.44 | 0.7287 | 1.764 | 3.396411 | 0.001863 | 0.0518196 |
| LOC409409 | 409409 | 9.55 | 5.65 | 23.40 | 27.70 | 4.0518 | 1.75 | 3.3635954 | 3.08E-11 | 2.75E-08 |
| LOC107965000 | 107965000 | 0.22 | 0.22 | 0.65 | 0.82 | -0.9483 | 1.734 | 3.3263764 | 0.003275 | 0.0718608 |
| LOC411353 | 411353 | 91.06 | 146.97 | 367.68 | 402.20 | 7.9773 | 1.6933 | 3.2339444 | 4.08E-11 | 3.08E-08 |
| LOC102654070 | 102654070 | 1.05 | 1.47 | 6.28 | 1.57 | 1.4132 | 1.6396 | 3.1156903 | 0.004622 | 0.0886532 |
| LOC408603 | 408603 | 75.70 | 18.08 | 73.34 | 218.65 | 6.5912 | 1.6386 | 3.1135787 | 0.003233 | 0.0716093 |
| LOC107964601 | 107964601 | 1.26 | 1.20 | 4.58 | 3.08 | 1.3645 | 1.6359 | 3.1079148 | 2.79E-05 | 0.0026637 |
| LOC102654792 | 102654792 | 0.65 | 0.20 | 1.49 | 1.08 | -0.1567 | 1.6203 | 3.0743953 | 0.005222 | 0.0956638 |
| LOC100577053 | 100577053 | 127.25 | 87.36 | 166.73 | 475.88 | 7.7434 | 1.5822 | 2.9941578 | 4.75E-05 | 0.0039194 |
| LOC408532 | 408532 | 17.78 | 12.67 | 51.62 | 38.91 | 4.9202 | 1.5728 | 2.9749059 | 8.68E-13 | 1.42E-09 |
| LOC107964603 | 107964603 | 0.48 | 1.01 | 2.30 | 2.13 | 0.6051 | 1.5614 | 2.9513399 | 0.000617 | 0.0249196 |
| LOC726655 | 726655 | 1.83 | 1.96 | 7.64 | 3.24 | 1.8982 | 1.5231 | 2.8740403 | 0.000468 | 0.0212828 |
| LOC408291 | 408291 | 1782.38 | 1323.93 | 3696.73 | 4946.24 | 11.52 | 1.4763 | 2.7823872 | 3.06E-09 | 1.25E-06 |
| LOC552832 | 552832 | 43.39 | 19.60 | 52.66 | 120.55 | 5.8836 | 1.4592 | 2.7495562 | 0.00017 | 0.0102047 |
| LOC102654496 | 102654496 | 3.77 | 1.87 | 4.40 | 10.72 | 2.3739 | 1.4222 | 2.6799733 | 0.002306 | 0.058123 |
| LOC100578833 | 100578833 | 6.13 | 4.76 | 15.39 | 13.70 | 3.3259 | 1.4177 | 2.6715704 | 6.86E-08 | 1.87E-05 |
| LOC724721 | 724721 | 139.35 | 133.54 | 302.16 | 418.75 | 7.9569 | 1.4014 | 2.6415994 | 1.17E-09 | 6.05E-07 |
| LOC107965522 | 107965522 | 1.48 | 1.99 | 2.83 | 6.23 | 1.6512 | 1.3762 | 2.5958149 | 0.001756 | 0.0495937 |
| LOC724570 | 724570 | 1.37 | 2.24 | 3.64 | 5.70 | 1.7039 | 1.365 | 2.5758109 | 0.000597 | 0.0244738 |
| LOC409881 | 409881 | 164.50 | 117.23 | 453.09 | 269.42 | 7.9721 | 1.3588 | 2.5647338 | 1.16E-06 | 0.000207 |
| LOC552066 | 552066 | 213.17 | 206.19 | 382.02 | 691.61 | 8.544 | 1.3562 | 2.560035 | 8.24E-07 | 0.0001526 |
| LOC406147 | 406147 | 13021.43 | 5804.92 | 18009.31 | 29958.84 | 14.027 | 1.3493 | 2.5479267 | 0.000137 | 0.0085181 |
| LOC410559 | 410559 | 5.76 | 7.59 | 18.32 | 15.54 | 3.5652 | 1.3423 | 2.5355585 | 9.30E-08 | 2.40E-05 |
| LOC102656485 | 102656485 | 0.54 | 0.66 | 1.36 | 1.67 | 0.1315 | 1.3245 | 2.5044537 | 0.003312 | 0.071952 |
| LOC100576758 | 100576758 | 2.02 | 3.46 | 7.67 | 6.00 | 2.2718 | 1.3161 | 2.4899086 | 0.000212 | 0.0118128 |
| LOC410828 | 410828 | 14.10 | 8.11 | 19.06 | 35.89 | 4.2695 | 1.3068 | 2.473897 | 4.43E-05 | 0.0036899 |
| LOC102654418 | 102654418 | 3.74 | 1.42 | 4.66 | 8.06 | 2.1637 | 1.3005 | 2.4631166 | 0.004978 | 0.0927393 |
| LOC102654095 | 102654095 | 3.87 | 3.17 | 12.46 | 4.85 | 2.6203 | 1.3003 | 2.4627997 | 0.001959 | 0.0528505 |
| LOC102653649 | 102653649 | 0.83 | 0.52 | 1.83 | 1.47 | 0.2718 | 1.2978 | 2.4586013 | 0.004606 | 0.088505 |
| LOC724216 | 724216 | 344.48 | 369.86 | 932.82 | 817.64 | 9.2673 | 1.293 | 2.4504553 | 1.70E-09 | 7.97E-07 |
| LOC551176 | 551176 | 80.09 | 77.64 | 282.01 | 101.90 | 7.0819 | 1.2835 | 2.4343432 | 0.000339 | 0.0167457 |
| LOC107965950 | 107965950 | 1.16 | 1.25 | 3.27 | 2.52 | 1.067 | 1.2665 | 2.4057861 | 0.00094 | 0.0329018 |
| LOC100578349 | 100578349 | 2.56 | 1.77 | 3.95 | 6.36 | 1.878 | 1.2511 | 2.3801829 | 0.001034 | 0.0348988 |
| LOC102656859 | 102656859 | 16.63 | 11.10 | 28.01 | 37.79 | 4.5482 | 1.2466 | 2.3728677 | 1.43E-07 | 3.36E-05 |
| LOC100576886 | 100576886 | 21.52 | 18.74 | 50.28 | 45.10 | 5.085 | 1.2449 | 2.3699691 | 2.39E-11 | 2.75E-08 |
| Jhe | Jhe | 670.31 | 317.03 | 812.78 | 1526.56 | 9.6999 | 1.2445 | 2.3693135 | 0.000493 | 0.02203 |
| LOC551369 | 551369 | 74.76 | 73.27 | 250.94 | 97.54 | 6.9564 | 1.2354 | 2.3545083 | 0.000283 | 0.0146376 |
| LOC113218518 | 113218518 | 16.54 | 15.35 | 30.15 | 43.89 | 4.7278 | 1.2138 | 2.319538 | 1.00E-08 | 3.39E-06 |
| LOC100578399 | 100578399 | 4.98 | 6.48 | 13.19 | 13.24 | 3.2488 | 1.204 | 2.3037903 | 5.22E-06 | 0.000684 |
| LOC727164 | 727164 | 1.51 | 3.14 | 5.05 | 5.67 | 1.955 | 1.1999 | 2.2971934 | 0.002302 | 0.058123 |
| Fabp | Fabp | 3618.03 | 3749.66 | 7349.96 | 9569.74 | 12.568 | 1.1994 | 2.2964655 | 1.44E-07 | 3.36E-05 |
| TpnCIIb | TpnCIIb | 47.35 | 38.36 | 129.91 | 64.24 | 6.1297 | 1.1801 | 2.2658506 | 5.41E-05 | 0.0041484 |
| LOC102656479 | 102656479 | 11.84 | 5.11 | 14.11 | 24.22 | 3.7892 | 1.1774 | 2.2617474 | 0.001345 | 0.0415355 |
| LOC411569 | 411569 | 87.22 | 42.39 | 91.43 | 199.08 | 6.7146 | 1.1644 | 2.2413277 | 0.001571 | 0.0457911 |
| LOC726247 | 726247 | 2.91 | 4.30 | 6.54 | 9.64 | 2.5528 | 1.1629 | 2.2390193 | 0.000597 | 0.0244738 |
| LOC408453 | 408453 | 201.04 | 202.28 | 383.88 | 517.73 | 8.3498 | 1.1605 | 2.2353554 | 3.07E-07 | 6.41E-05 |
| LOC410296 | 410296 | 34.22 | 31.76 | 48.84 | 96.89 | 5.726 | 1.1426 | 2.2078364 | 3.60E-05 | 0.0032461 |
| LOC411633 | 411633 | 102.12 | 93.28 | 259.11 | 169.95 | 7.2869 | 1.1349 | 2.1960037 | 1.92E-06 | 0.000315 |
| LOC412203 | 412203 | 1.48 | 1.33 | 3.80 | 2.33 | 1.1905 | 1.1298 | 2.1883076 | 0.005163 | 0.0951186 |
| LOC411978 | 411978 | 51.22 | 37.28 | 79.49 | 114.06 | 6.1401 | 1.1288 | 2.1866966 | 2.88E-06 | 0.0004288 |
| LOC726262 | 726262 | 5.14 | 5.55 | 8.77 | 14.65 | 3.0946 | 1.1285 | 2.1862676 | 0.000258 | 0.0137566 |
| LOC113218574 | 113218574 | 41.97 | 27.34 | 39.71 | 110.39 | 5.7772 | 1.1145 | 2.1652467 | 0.002827 | 0.066565 |
| LOC410058 | 410058 | 87.59 | 70.51 | 228.19 | 100.43 | 6.9277 | 1.0558 | 2.0788324 | 0.000903 | 0.032228 |
| LOC100576637 | 100576637 | 98.43 | 107.67 | 131.84 | 295.68 | 7.3075 | 1.0524 | 2.0739067 | 0.000743 | 0.0277513 |
| LOC408827 | 408827 | 62.79 | 93.58 | 158.20 | 159.82 | 6.8903 | 1.024 | 2.033513 | 7.95E-06 | 0.0009762 |
| LOC410492 | 410492 | 104.35 | 89.50 | 156.00 | 237.50 | 7.1982 | 1.0212 | 2.0296676 | 2.13E-05 | 0.0021598 |
| LOC551448 | 551448 | 242.92 | 206.09 | 370.14 | 541.14 | 8.4098 | 1.0211 | 2.0294196 | 2.25E-05 | 0.0022556 |
| LOC410635 | 410635 | 136.10 | 119.37 | 211.91 | 306.43 | 7.5959 | 1.0207 | 2.0288544 | 1.30E-05 | 0.0014661 |

**Supplementary Table 6.** List of downregulated genes identified in the DWV-infected honey bee larvae library

| **Gene symbol** | **Gene ID** | **CPM**  **Control_1** | **CPM**  **Control_2** | **CPM**  **infected_1** | **CPM**  **infected_2** | **logCPM** | **logFC** | **Fold change** | **PValue** | **FDR** |
| --- | --- | --- | --- | --- | --- | --- | --- | --- | --- | --- |
| LOC107963975 | 107963975 | 61.39 | 12.21 | 0.89 | 1.61 | 4.256252 | -4.8853 | -29.554237 | 2.43E-15 | 7.95E-12 |
| LOC102655555 | 102655555 | 1.10 | 0.34 | 0.03 | 0.07 | -1.16059 | -3.9282 | -15.222908 | 5.38E-05 | 0.004148 |
| LOC413908 | 413908 | 25.42 | 5.67 | 2.25 | 0.07 | 3.077731 | -3.732 | -13.28736 | 0.0001046 | 0.006819 |
| LOC100576697 | 100576697 | 2.02 | 1.13 | 0.13 | 0.10 | -0.12263 | -3.7212 | -13.188382 | 5.55E-09 | 2.02E-06 |
| LOC724565 | 724565 | 32.01 | 21.52 | 3.48 | 1.34 | 3.876929 | -3.4561 | -10.974609 | 2.38E-20 | 2.34E-16 |
| LOC409751 | 409751 | 13.29 | 4.03 | 1.57 | 0.29 | 2.287812 | -3.1926 | -9.1426332 | 2.30E-06 | 0.00037 |
| LOC100577819 | 100577819 | 0.46 | 0.34 | 0.05 | 0.03 | -1.81419 | -3.1026 | -8.5897906 | 0.00029576 | 0.014971 |
| LOC102656882 | 102656882 | 4.57 | 1.30 | 0.31 | 0.43 | 0.786328 | -2.9837 | -7.9103733 | 3.30E-06 | 0.000477 |
| Est-6 | Est-6 | 0.46 | 0.86 | 0.08 | 0.10 | -1.17309 | -2.8678 | -7.2993657 | 0.00016542 | 0.009966 |
| LOC113218894 | 113218894 | 5.84 | 0.39 | 0.39 | 0.46 | 0.875184 | -2.8622 | -7.2710392 | 0.00194814 | 0.052702 |
| LOC102655781 | 102655781 | 1.24 | 1.87 | 0.24 | 0.20 | -0.05173 | -2.8181 | -7.0524829 | 1.98E-07 | 4.22E-05 |
| LOC410850 | 410850 | 0.30 | 0.34 | 0.00 | 0.10 | -2.03318 | -2.7541 | -6.7464724 | 0.0048884 | 0.091629 |
| LOC410317 | 410317 | 5.11 | 8.20 | 1.26 | 0.85 | 1.984337 | -2.6457 | -6.2579832 | 4.76E-11 | 3.34E-08 |
| LOC725087 | 725087 | 1.29 | 0.42 | 0.18 | 0.10 | -0.83175 | -2.5411 | -5.8202981 | 0.00065755 | 0.025597 |
| LOC410405 | 410405 | 5.33 | 2.55 | 0.52 | 0.88 | 1.260743 | -2.4904 | -5.6195071 | 6.07E-07 | 0.000117 |
| LOC102656658 | 102656658 | 0.81 | 1.01 | 0.16 | 0.16 | -0.7261 | -2.4782 | -5.5721457 | 3.15E-05 | 0.002919 |
| LOC410406 | 410406 | 1.67 | 2.24 | 0.29 | 0.43 | 0.302263 | -2.4583 | -5.4956416 | 3.53E-07 | 7.07E-05 |
| LOC100576130 | 100576130 | 0.97 | 0.74 | 0.18 | 0.13 | -0.80378 | -2.3941 | -5.2566035 | 0.00010269 | 0.006819 |
| LOC724900 | 724900 | 6.99 | 6.80 | 2.22 | 0.49 | 2.079763 | -2.3248 | -5.0099813 | 7.62E-06 | 0.000947 |
| LOC102654839 | 102654839 | 1.21 | 0.54 | 0.21 | 0.13 | -0.76573 | -2.3133 | -4.9702859 | 0.00065947 | 0.025597 |
| LOC726459 | 726459 | 0.94 | 0.93 | 0.24 | 0.13 | -0.66319 | -2.3031 | -4.9350638 | 7.67E-05 | 0.005535 |
| crh-BP | crh-BP | 0.40 | 0.44 | 0.13 | 0.03 | -1.65536 | -2.2543 | -4.7709607 | 0.0027627 | 0.066073 |
| LOC727193 | 727193 | 65.53 | 22.42 | 9.82 | 8.75 | 4.739424 | -2.2429 | -4.733389 | 2.27E-09 | 9.68E-07 |
| LOC113219002 | 113219002 | 0.40 | 0.42 | 0.13 | 0.03 | -1.6857 | -2.2114 | -4.6312578 | 0.00350549 | 0.073871 |
| LOC551044 | 551044 | 15.60 | 29.87 | 5.99 | 3.90 | 3.802129 | -2.196 | -4.5819156 | 2.89E-11 | 2.75E-08 |
| LOC411894 | 411894 | 1.40 | 1.23 | 0.29 | 0.29 | -0.19515 | -2.1592 | -4.4666279 | 2.53E-05 | 0.002455 |
| LOC413346 | 413346 | 22.41 | 29.30 | 11.15 | 1.54 | 4.019114 | -2.0224 | -4.0625906 | 0.00053236 | 0.023158 |
| LOC100577043 | 100577043 | 75.84 | 114.09 | 35.39 | 11.80 | 5.892142 | -2.0074 | -4.0204848 | 3.78E-07 | 7.43E-05 |
| LOC725264 | 725264 | 3.47 | 6.46 | 1.54 | 0.92 | 1.675318 | -2.0002 | -4.0006313 | 4.57E-06 | 0.000615 |
| LOC725288 | 725288 | 1.29 | 0.84 | 0.24 | 0.29 | -0.45468 | -1.9961 | -3.9890789 | 0.00043125 | 0.020166 |
| LOC107965335 | 107965335 | 0.43 | 0.96 | 0.16 | 0.20 | -0.98741 | -1.9685 | -3.9137316 | 0.00384353 | 0.078754 |
| LOC100577334 | 100577334 | 0.59 | 0.66 | 0.13 | 0.20 | -1.12132 | -1.9392 | -3.8348764 | 0.00213296 | 0.054975 |
| LOC726896 | 726896 | 1.35 | 1.87 | 0.44 | 0.39 | 0.122938 | -1.9262 | -3.8005574 | 5.33E-05 | 0.004148 |
| LOC409327 | 409327 | 0.91 | 0.64 | 0.18 | 0.23 | -0.84963 | -1.9029 | -3.7395429 | 0.00130649 | 0.04099 |
| LOC102655737 | 102655737 | 1.21 | 0.59 | 0.16 | 0.33 | -0.6632 | -1.9023 | -3.7381024 | 0.00355131 | 0.074676 |
| 5-HT2alpha | 5-HT2alpha | 0.86 | 0.98 | 0.29 | 0.20 | -0.61686 | -1.8956 | -3.7208039 | 0.00057264 | 0.024079 |
| LOC410001 | 410001 | 1.43 | 0.91 | 0.44 | 0.16 | -0.30963 | -1.8895 | -3.705072 | 0.0012262 | 0.039351 |
| NLG-4 | NLG-4 | 0.51 | 0.74 | 0.24 | 0.10 | -1.10678 | -1.8439 | -3.5896779 | 0.00349258 | 0.073782 |
| LOC102656453 | 102656453 | 0.89 | 0.69 | 0.18 | 0.26 | -0.81481 | -1.822 | -3.5357295 | 0.00152199 | 0.045018 |
| LOC100577019 | 100577019 | 4.22 | 5.58 | 1.73 | 1.05 | 1.693223 | -1.8069 | -3.4988994 | 2.63E-06 | 0.000397 |
| LOC408804 | 408804 | 1.56 | 1.08 | 0.37 | 0.39 | -0.12442 | -1.7887 | -3.4549697 | 0.00039983 | 0.018984 |
| Y-y | Y-y | 1.37 | 1.11 | 0.47 | 0.23 | -0.207 | -1.7829 | -3.4410812 | 0.00059333 | 0.024474 |
| LOC410291 | 410291 | 1.67 | 1.72 | 0.50 | 0.49 | 0.22265 | -1.7688 | -3.4077361 | 6.08E-05 | 0.00452 |
| LOC412774 | 412774 | 1.53 | 1.38 | 0.31 | 0.56 | 0.016803 | -1.7529 | -3.370392 | 0.00038298 | 0.018436 |
| LOC550964 | 550964 | 1.35 | 0.66 | 0.39 | 0.20 | -0.48227 | -1.7286 | -3.3141226 | 0.00484736 | 0.09119 |
| LOC411213 | 411213 | 1.10 | 0.74 | 0.29 | 0.26 | -0.59421 | -1.7217 | -3.2983299 | 0.00191578 | 0.052258 |
| LOC107965485 | 107965485 | 15.90 | 17.34 | 5.13 | 4.95 | 3.448676 | -1.7204 | -3.295233 | 2.90E-12 | 4.06E-09 |
| LOC113218794 | 113218794 | 0.70 | 0.64 | 0.26 | 0.13 | -1.00211 | -1.7169 | -3.2873123 | 0.00531101 | 0.096761 |
| LOC113219060 | 113219060 | 1.35 | 1.03 | 0.42 | 0.29 | -0.2487 | -1.7092 | -3.2697829 | 0.00066458 | 0.025693 |
| LOC411009 | 411009 | 3.63 | 3.27 | 0.97 | 1.15 | 1.219639 | -1.7064 | -3.2635313 | 6.50E-06 | 0.00083 |
| LOC725649 | 725649 | 1.00 | 1.42 | 0.18 | 0.59 | -0.21149 | -1.6745 | -3.1921606 | 0.00452909 | 0.087897 |
| LOC726476 | 726476 | 52.40 | 68.72 | 24.58 | 13.93 | 5.322243 | -1.6511 | -3.1406431 | 2.05E-10 | 1.26E-07 |
| LOC724225 | 724225 | 1.05 | 0.76 | 0.37 | 0.20 | -0.59757 | -1.6446 | -3.1267138 | 0.00370599 | 0.076616 |
| LOC412273 | 412273 | 143.63 | 163.72 | 53.68 | 45.07 | 6.667029 | -1.6376 | -3.1113924 | 4.82E-15 | 1.18E-11 |
| LOC102655014 | 102655014 | 0.91 | 1.33 | 0.44 | 0.26 | -0.30292 | -1.6365 | -3.109213 | 0.00190943 | 0.05223 |
| LOC102655278 | 102655278 | 1.18 | 0.74 | 0.26 | 0.36 | -0.51882 | -1.6249 | -3.0842576 | 0.00420118 | 0.083513 |
| LOC107964173 | 107964173 | 1.29 | 1.25 | 0.29 | 0.56 | -0.13287 | -1.6069 | -3.0458936 | 0.00167712 | 0.047876 |
| LOC406144 | 406144 | 206.18 | 177.92 | 84.26 | 42.81 | 6.998699 | -1.5952 | -3.0213859 | 3.12E-08 | 9.57E-06 |
| LOC102655819 | 102655819 | 7.59 | 11.84 | 3.48 | 2.95 | 2.712626 | -1.5922 | -3.0151671 | 1.14E-06 | 0.000207 |
| LOC408554 | 408554 | 1.99 | 2.09 | 0.58 | 0.79 | 0.516985 | -1.588 | -3.0063187 | 0.00021009 | 0.011789 |
| LOC413428 | 413428 | 11.06 | 11.00 | 4.29 | 3.02 | 2.893364 | -1.5878 | -3.0058088 | 7.52E-08 | 2.00E-05 |
| LOC726441 | 726441 | 0.89 | 0.86 | 0.34 | 0.23 | -0.62824 | -1.5874 | -3.0050004 | 0.00329301 | 0.071861 |
| LOC107965965 | 107965965 | 3.04 | 1.84 | 1.02 | 0.59 | 0.763108 | -1.5801 | -2.98997 | 0.00065705 | 0.025597 |
| LOC411209 | 411209 | 1.37 | 1.30 | 0.52 | 0.36 | -0.05791 | -1.5741 | -2.9774891 | 0.00097109 | 0.033696 |
| LOC410515 | 410515 | 12.24 | 7.39 | 3.19 | 3.44 | 2.731637 | -1.5643 | -2.9572972 | 2.55E-06 | 0.000397 |
| LOC102655639 | 102655639 | 4.49 | 7.27 | 2.72 | 1.25 | 2.009778 | -1.5572 | -2.9429218 | 0.00027002 | 0.014333 |
| LOC100577073 | 100577073 | 1.26 | 1.08 | 0.52 | 0.26 | -0.22837 | -1.5426 | -2.9132862 | 0.00251887 | 0.061378 |
| LOC412855 | 412855 | 1.53 | 1.45 | 0.50 | 0.52 | 0.097784 | -1.5409 | -2.9096923 | 0.00073006 | 0.027468 |
| Y-e3 | Y-e3 | 22.38 | 20.61 | 9.08 | 5.70 | 3.861332 | -1.5346 | -2.8970507 | 1.81E-09 | 8.10E-07 |
| LOC409598 | 409598 | 5.46 | 3.98 | 1.65 | 1.61 | 1.699907 | -1.5325 | -2.8928191 | 1.77E-05 | 0.001884 |
| LOC100577636 | 100577636 | 1.02 | 0.96 | 0.39 | 0.29 | -0.44494 | -1.5038 | -2.8358744 | 0.00327516 | 0.071861 |
| LOC412887 | 412887 | 1.40 | 1.13 | 0.31 | 0.59 | -0.11898 | -1.4982 | -2.8249934 | 0.0034504 | 0.073499 |
| LOC410624 | 410624 | 2.58 | 3.93 | 1.47 | 0.82 | 1.19228 | -1.4974 | -2.8232778 | 0.00053188 | 0.023158 |
| LOC113218814 | 113218814 | 2.77 | 1.20 | 0.65 | 0.75 | 0.494805 | -1.4927 | -2.8141863 | 0.00405185 | 0.081336 |
| LOC411053 | 411053 | 62.01 | 74.20 | 28.98 | 19.37 | 5.530846 | -1.4927 | -2.8140653 | 3.49E-11 | 2.86E-08 |
| LOC726068 | 726068 | 3.39 | 2.33 | 1.15 | 0.88 | 1.009438 | -1.4791 | -2.7878385 | 0.00034574 | 0.016976 |
| LOC410925 | 410925 | 1.32 | 1.52 | 0.52 | 0.49 | 0.049172 | -1.4771 | -2.783944 | 0.00113556 | 0.037295 |
| LOC100577879 | 100577879 | 19.13 | 15.38 | 8.72 | 3.67 | 3.562444 | -1.4717 | -2.7735719 | 2.30E-05 | 0.002286 |
| LOC100576976 | 100576976 | 1.35 | 1.45 | 0.52 | 0.49 | 0.031591 | -1.4527 | -2.7372159 | 0.00135387 | 0.04159 |
| LOC102655167 | 102655167 | 1.64 | 1.89 | 0.92 | 0.36 | 0.357144 | -1.4398 | -2.7127552 | 0.00337045 | 0.072371 |
| LOC409510 | 409510 | 2.13 | 1.99 | 0.63 | 0.92 | 0.568191 | -1.4199 | -2.6756057 | 0.00097706 | 0.033784 |
| LOC100576222 | 100576222 | 2.04 | 1.30 | 0.65 | 0.59 | 0.280566 | -1.4162 | -2.6687962 | 0.00234656 | 0.058634 |
| LOC100577135 | 100577135 | 28.97 | 24.17 | 11.91 | 7.96 | 4.196963 | -1.415 | -2.6665345 | 3.18E-09 | 1.25E-06 |
| LOC550828 | 550828 | 102.77 | 96.43 | 47.56 | 28.94 | 6.108815 | -1.3794 | -2.6016381 | 1.76E-08 | 5.76E-06 |
| LOC410539 | 410539 | 157.21 | 112.76 | 48.32 | 56.51 | 6.551146 | -1.365 | -2.5757345 | 1.54E-09 | 7.54E-07 |
| LOC725725 | 725725 | 33.12 | 34.36 | 4.71 | 21.57 | 4.554697 | -1.3626 | -2.5714996 | 0.00358227 | 0.075166 |
| LOC410894 | 410894 | 759.84 | 1052.73 | 469.00 | 238.58 | 9.299523 | -1.357 | -2.5614657 | 9.80E-06 | 0.001146 |
| LOC100576471 | 100576471 | 2.10 | 2.04 | 0.65 | 0.98 | 0.594983 | -1.3454 | -2.5410196 | 0.00161569 | 0.046665 |
| LOC107964039 | 107964039 | 3.01 | 2.80 | 1.20 | 1.08 | 1.069166 | -1.3407 | -2.532829 | 0.00039907 | 0.018984 |
| LOC102655409 | 102655409 | 2.64 | 3.61 | 1.62 | 0.82 | 1.173952 | -1.3403 | -2.5320611 | 0.00200872 | 0.053855 |
| LOC102653655 | 102653655 | 11.86 | 6.21 | 2.75 | 4.49 | 2.676484 | -1.3231 | -2.5021028 | 0.00061918 | 0.02492 |
| LOC724269 | 724269 | 3.98 | 2.43 | 1.15 | 1.44 | 1.212593 | -1.3066 | -2.4736466 | 0.00155196 | 0.045493 |
| LOC725434 | 725434 | 13.13 | 12.82 | 6.26 | 4.23 | 3.200618 | -1.3024 | -2.4663667 | 3.55E-06 | 0.000506 |
| LOC551761 | 551761 | 80.60 | 125.21 | 54.18 | 29.53 | 6.179499 | -1.297 | -2.4571385 | 1.22E-05 | 0.001398 |
| LOC551137 | 551137 | 2.66 | 1.89 | 0.65 | 1.21 | 0.737485 | -1.2964 | -2.4562086 | 0.00454697 | 0.087905 |
| LOC100577472 | 100577472 | 2.61 | 2.90 | 1.31 | 0.92 | 1.007677 | -1.2955 | -2.4545547 | 0.00104986 | 0.035067 |
| LOC410235 | 410235 | 2.47 | 3.32 | 1.18 | 1.18 | 1.079083 | -1.2952 | -2.4541096 | 0.00082506 | 0.030008 |
| LOC410304 | 410304 | 4.57 | 4.67 | 1.52 | 2.29 | 1.736986 | -1.2837 | -2.4346067 | 0.00031715 | 0.01589 |
| LOC408451 | 408451 | 12.19 | 7.47 | 3.77 | 4.36 | 2.810523 | -1.2738 | -2.4179123 | 9.56E-05 | 0.006478 |
| LOC113219095 | 113219095 | 1.78 | 2.14 | 0.89 | 0.72 | 0.540254 | -1.2711 | -2.4134294 | 0.00232525 | 0.05825 |
| LOC412763 | 412763 | 18.32 | 16.73 | 7.25 | 7.34 | 3.642473 | -1.2636 | -2.4009737 | 3.05E-08 | 9.57E-06 |
| LOC113218797 | 113218797 | 3.36 | 2.16 | 0.99 | 1.31 | 1.015845 | -1.2626 | -2.3991973 | 0.00266804 | 0.064692 |
| LOC408908 | 408908 | 4.17 | 4.94 | 2.30 | 1.47 | 1.724059 | -1.26 | -2.3949824 | 0.00050655 | 0.022407 |
| LOC411158 | 411158 | 15.98 | 20.41 | 7.33 | 8.00 | 3.701621 | -1.2486 | -2.3760591 | 1.56E-07 | 3.57E-05 |
| LOC408471 | 408471 | 2.56 | 2.41 | 1.10 | 0.98 | 0.874261 | -1.2465 | -2.372618 | 0.00130094 | 0.040946 |
| LOC724736 | 724736 | 2.18 | 2.90 | 0.97 | 1.18 | 0.909409 | -1.2447 | -2.3696379 | 0.00217325 | 0.055722 |
| LOC408896 | 408896 | 4.68 | 5.40 | 2.04 | 2.23 | 1.873553 | -1.2406 | -2.3629651 | 0.00017838 | 0.010489 |
| LOC411693 | 411693 | 1.86 | 1.79 | 0.68 | 0.88 | 0.453969 | -1.2255 | -2.3383861 | 0.00370543 | 0.076616 |
| LOC408970 | 408970 | 114.28 | 127.10 | 60.88 | 42.84 | 6.432365 | -1.2179 | -2.3260854 | 5.47E-08 | 1.58E-05 |
| LOC107963995 | 107963995 | 5.33 | 7.00 | 2.96 | 2.33 | 2.165184 | -1.2176 | -2.3256343 | 0.00022434 | 0.012258 |
| LOC412778 | 412778 | 3.95 | 3.41 | 1.28 | 1.90 | 1.435517 | -1.2166 | -2.3239126 | 0.00121269 | 0.039045 |
| LOC411079 | 411079 | 8.61 | 6.93 | 3.25 | 3.44 | 2.492778 | -1.2155 | -2.3222373 | 5.60E-05 | 0.004229 |
| LOC552792 | 552792 | 2.29 | 2.97 | 1.18 | 1.08 | 0.966537 | -1.2153 | -2.3218403 | 0.00210593 | 0.054722 |
| LOC724803 | 724803 | 19.45 | 18.03 | 8.45 | 7.70 | 3.753633 | -1.2123 | -2.3170141 | 5.65E-08 | 1.59E-05 |
| LOC107965483 | 107965483 | 2.13 | 1.84 | 0.92 | 0.79 | 0.572503 | -1.2115 | -2.3157114 | 0.00292014 | 0.068276 |
| LOC552048 | 552048 | 46.43 | 47.77 | 22.59 | 18.09 | 5.079197 | -1.2098 | -2.3130102 | 8.97E-11 | 5.87E-08 |
| LOC102654084 | 102654084 | 2.04 | 2.68 | 0.99 | 1.05 | 0.818562 | -1.2084 | -2.3108553 | 0.00268419 | 0.064923 |
| LOC100576186 | 100576186 | 10.30 | 8.15 | 4.37 | 3.61 | 2.741074 | -1.2065 | -2.3077914 | 5.02E-05 | 0.004106 |
| LOC411212 | 411212 | 6.24 | 6.04 | 2.54 | 2.79 | 2.162186 | -1.2064 | -2.3076237 | 0.00010304 | 0.006819 |
| LOC724640 | 724640 | 1.86 | 2.43 | 0.94 | 0.92 | 0.686508 | -1.2027 | -2.3016526 | 0.00333479 | 0.072149 |
| Mrjp7 | Mrjp7 | 17.94 | 17.90 | 7.30 | 8.33 | 3.694398 | -1.199 | -2.2958081 | 1.22E-07 | 2.98E-05 |
| LOC100577530 | 100577530 | 15.87 | 10.66 | 6.02 | 5.54 | 3.262572 | -1.1966 | -2.2919734 | 2.43E-05 | 0.002387 |
| LOC726283 | 726283 | 3.66 | 3.29 | 1.15 | 1.90 | 1.359527 | -1.1947 | -2.2889882 | 0.00215263 | 0.055337 |
| LOC107964623 | 107964623 | 9.39 | 17.61 | 8.56 | 3.21 | 3.291779 | -1.1933 | -2.2868221 | 0.00496789 | 0.092739 |
| LOC413380 | 413380 | 6.05 | 5.23 | 1.91 | 3.05 | 2.045322 | -1.1919 | -2.2845935 | 0.00070906 | 0.026884 |
| LOC726252 | 726252 | 48.48 | 35.29 | 16.67 | 20.03 | 4.915995 | -1.1914 | -2.2836791 | 8.79E-09 | 3.08E-06 |
| LOC102654056 | 102654056 | 2.45 | 2.38 | 0.92 | 1.21 | 0.853485 | -1.187 | -2.2767401 | 0.00304528 | 0.069708 |
| LOC410465 | 410465 | 4.49 | 3.88 | 2.54 | 1.15 | 1.63058 | -1.1706 | -2.2510789 | 0.00488935 | 0.091629 |
| nAChRa9 | nAChRa9 | 146.05 | 205.72 | 109.99 | 46.58 | 6.990764 | -1.1674 | -2.2461193 | 0.00050426 | 0.022406 |
| CPR16 | CPR16 | 3.44 | 4.25 | 1.73 | 1.70 | 1.514136 | -1.1634 | -2.2398722 | 0.00104742 | 0.035067 |
| LOC727284 | 727284 | 6.03 | 6.31 | 3.85 | 1.64 | 2.183731 | -1.1594 | -2.2336625 | 0.00375585 | 0.077322 |
| LOC413274 | 413274 | 2.66 | 3.12 | 1.31 | 1.28 | 1.114591 | -1.1585 | -2.2322453 | 0.00206972 | 0.054199 |
| LOC552672 | 552672 | 16.89 | 17.83 | 9.68 | 5.87 | 3.661774 | -1.1544 | -2.2259232 | 1.76E-05 | 0.001884 |
| LOC102654867 | 102654867 | 27.63 | 17.90 | 10.91 | 9.60 | 4.052027 | -1.1482 | -2.2162987 | 3.19E-06 | 0.000468 |
| LOC107964010 | 107964010 | 4.55 | 3.32 | 1.78 | 1.77 | 1.547103 | -1.1443 | -2.2104578 | 0.00154223 | 0.045343 |
| LOC100576196 | 100576196 | 8.31 | 8.47 | 4.21 | 3.41 | 2.627959 | -1.1351 | -2.1963609 | 0.0001048 | 0.006819 |
| LOC551628 | 551628 | 4.17 | 3.93 | 2.30 | 1.38 | 1.596213 | -1.1273 | -2.1845607 | 0.00251374 | 0.061378 |
| LOC551597 | 551597 | 5.38 | 3.22 | 2.28 | 1.64 | 1.678149 | -1.1266 | -2.183447 | 0.00401413 | 0.080942 |
| LOC100577752 | 100577752 | 5.70 | 3.51 | 2.15 | 2.06 | 1.776119 | -1.1265 | -2.1832092 | 0.00243735 | 0.060289 |
| LOC100577385 | 100577385 | 3.17 | 2.73 | 1.20 | 1.51 | 1.150356 | -1.1244 | -2.1800578 | 0.00295664 | 0.068639 |
| LOC410920 | 410920 | 7.24 | 3.71 | 2.91 | 2.13 | 2.023621 | -1.1138 | -2.1642058 | 0.00526491 | 0.096099 |
| LOC408797 | 408797 | 26.50 | 20.16 | 12.25 | 9.41 | 4.100839 | -1.1046 | -2.1503758 | 1.71E-06 | 0.000289 |
| LOC725046 | 725046 | 7.34 | 4.49 | 2.72 | 2.79 | 2.139034 | -1.102 | -2.1465235 | 0.00171927 | 0.048795 |
| LOC102654777 | 102654777 | 90.61 | 112.15 | 71.14 | 23.47 | 6.218019 | -1.0988 | -2.1418288 | 0.00384949 | 0.078754 |
| LOC725668 | 725668 | 3.74 | 5.01 | 2.46 | 1.61 | 1.71615 | -1.098 | -2.1405874 | 0.00307092 | 0.069807 |
| LOC410689 | 410689 | 7.42 | 6.14 | 3.53 | 2.82 | 2.337658 | -1.0897 | -2.128348 | 0.00048367 | 0.021888 |
| LOC113219265 | 113219265 | 1021.89 | 2232.73 | 903.87 | 626.85 | 10.22452 | -1.0883 | -2.126182 | 0.00098567 | 0.033801 |
| LOC724552 | 724552 | 47.56 | 66.71 | 32.74 | 21.01 | 5.39564 | -1.0867 | -2.1238971 | 8.91E-06 | 0.001067 |
| AChE-2 | AChE-2 | 47.70 | 45.83 | 18.11 | 25.99 | 5.107639 | -1.0862 | -2.1231123 | 1.66E-07 | 3.63E-05 |
| LOC102654344 | 102654344 | 4.14 | 5.01 | 2.62 | 1.67 | 1.783022 | -1.0856 | -2.1223265 | 0.00272263 | 0.06553 |
| LOC100578220 | 100578220 | 3.17 | 3.17 | 1.70 | 1.28 | 1.265976 | -1.0818 | -2.116623 | 0.00314158 | 0.070687 |
| LOC551736 | 551736 | 3.71 | 3.17 | 1.83 | 1.41 | 1.380434 | -1.078 | -2.1111387 | 0.00329281 | 0.071861 |
| LOC102654076 | 102654076 | 22.92 | 28.74 | 17.69 | 6.88 | 4.259597 | -1.0685 | -2.0972515 | 0.00180133 | 0.05054 |
| LOC409950 | 409950 | 6.94 | 5.62 | 3.04 | 2.95 | 2.235547 | -1.0677 | -2.0961408 | 0.0006443 | 0.025451 |
| LOC411248 | 411248 | 2.64 | 2.87 | 1.26 | 1.38 | 1.073972 | -1.0661 | -2.0938216 | 0.00429866 | 0.084595 |
| 18-w | 18-w | 3.09 | 3.17 | 1.20 | 1.80 | 1.252624 | -1.0655 | -2.0928892 | 0.00532173 | 0.096777 |
| LOC412215 | 412215 | 20.42 | 21.20 | 10.13 | 9.83 | 3.951571 | -1.0593 | -2.0838859 | 3.27E-07 | 6.69E-05 |
| LOC725964 | 725964 | 42.02 | 55.93 | 22.85 | 24.48 | 5.185865 | -1.0496 | -2.0699876 | 1.11E-07 | 2.80E-05 |
| LOC102654510 | 102654510 | 10.87 | 9.26 | 5.42 | 4.29 | 2.913915 | -1.0477 | -2.0671706 | 0.00024382 | 0.013156 |
| LOC409782 | 409782 | 7.56 | 7.32 | 3.38 | 3.83 | 2.483861 | -1.046 | -2.0648597 | 0.00036417 | 0.01778 |
| LOC100578680 | 100578680 | 17.70 | 14.54 | 7.56 | 8.10 | 3.590654 | -1.0418 | -2.0588315 | 1.17E-05 | 0.001356 |
| LOC410259 | 410259 | 3.87 | 5.43 | 1.88 | 2.65 | 1.820095 | -1.0404 | -2.0567484 | 0.00441334 | 0.086505 |
| LOC410362 | 410362 | 5.65 | 5.99 | 3.06 | 2.65 | 2.142549 | -1.0228 | -2.0319103 | 0.00098622 | 0.033801 |
| LOC410009 | 410009 | 10.81 | 10.34 | 5.10 | 5.31 | 2.993746 | -1.0224 | -2.0312827 | 0.00012976 | 0.008184 |
| LOC411021 | 411021 | 10.41 | 19.38 | 8.85 | 5.83 | 3.486448 | -1.0186 | -2.0259598 | 0.001895 | 0.052051 |
| LOC725754 | 725754 | 9.28 | 8.87 | 4.66 | 4.29 | 2.776088 | -1.0176 | -2.0245103 | 0.00028158 | 0.01463 |
| LOC410231 | 410231 | 6.75 | 7.81 | 3.98 | 3.21 | 2.463837 | -1.0147 | -2.0205055 | 0.00081895 | 0.030008 |
| LOC102654427 | 102654427 | 23.70 | 17.71 | 10.00 | 10.69 | 3.962881 | -1.0013 | -2.0018072 | 9.63E-06 | 0.001139 |

**Supplementary Table 7.** Gene ontology of upregulated genes

| **GO category** | **GO term (GO_ID)** | **No. of DEG** |
| --- | --- | --- |
| Molecular Function | Catalytic activity (GO:0003824) | 17 |
|  | Binding (GO:0005488) | 9 |
|  | Molecular function regulator (GO:0098772) | 3 |
|  | Molecular transducer activity (GO:0060089) | 2 |
|  | Transporter activity (GO:0005215) | 2 |
|  | Structural molecule activity (GO:0005198) | 1 |
| Biological Process | Cellular process (GO:0009987) | 19 |
|  | Metabolic process (GO:0008152) | 11 |
|  | Biological regulation (GO:0065007) | 7 |
|  | Response to stimulus (GO:0050896) | 2 |
|  | Signaling (GO:0023052) | 2 |
|  | Localization (GO:0051179) | 2 |
|  | Multicellular organismal process (GO:0032501) | 1 |
| Cellular Component | Cellular anatomical entity (GO:0110165) | 23 |
|  | Intracellular (GO:0005622) | 17 |
|  | Protein-containing complex (GO:0032991) | 4 |

**Supplementary Table 8.** Gene ontology of downregulated genes

| **GO category** | **GO term (GO_ID)** | **No. of DEG** |
| --- | --- | --- |
| Molecular Function | Catalytic activity (GO:0003824) | 19 |
|  | Binding (GO:0005488) | 17 |
|  | Molecular transducer activity (GO:0060089) | 4 |
|  | Molecular function regulator (GO:0098772) | 4 |
|  | Transporter activity (GO:0005215) | 2 |
|  | Structural molecule activity (GO:0005198) | 1 |
| Biological Process | Cellular process (GO:0009987) | 23 |
|  | Metabolic process (GO:0008152) | 15 |
|  | Biological regulation (GO:0065007) | 12 |
|  | Multicellular organismal process (GO:0032501) | 10 |
|  | Response to stimulus (GO:0050896) | 9 |
|  | Developmental process (GO:0032502) | 7 |
|  | Localization (GO:0051179) | 6 |
|  | Signaling (GO:0023052) | 5 |
|  | Biological adhesion (GO:0022610) | 4 |
|  | Locomotion (GO:0040011) | 3 |
| Cellular Component | Cellular anatomical entity (GO:0110165) | 41 |
|  | Intracellular (GO:0005622) | 11 |
|  | Protein-containing complex (GO:0032991) | 2 |

**Supplementary Table 9.** KEGG pathway hit results of upregulated genes

| ko01100 Metabolic pathways (13) | | | |  | |  |  | |  |
| --- | --- | --- | --- | --- | --- | --- | --- | --- | --- |
|  | ko:K00010 iolG; myo-inositol 2-dehydrogenase / D-chiro-inositol 1-dehydrogenase [EC:1.1.1.18 1.1.1.369] | | | | | | | | |
|  | ko:K00108 betA; choline dehydrogenase [EC:1.1.99.1] | | | | | |  | |  |
|  | ko:K00134 GAPDH; glyceraldehyde 3-phosphate dehydrogenase [EC:1.2.1.12] | | | | | | | | |
|  | ko:K00281 GLDC; glycine dehydrogenase [EC:1.4.4.2] | | | | | |  | |  |
|  | ko:K00430 E1.11.1.7; peroxidase [EC:1.11.1.7] | | | | |  |  | |  |
|  | ko:K00453 TDO2; tryptophan 2,3-dioxygenase [EC:1.13.11.11] | | | | | |  | |  |
|  | ko:K00552 GNMT; glycine N-methyltransferase [EC:2.1.1.20] | | | | | |  | |  |
|  | ko:K00948 PRPS; ribose-phosphate pyrophosphokinase [EC:2.7.6.1] | | | | | |  | |  |
|  | ko:K01672 CA; carbonic anhydrase [EC:4.2.1.1] | | | | |  |  | |  |
|  | ko:K01784 galE; UDP-glucose 4-epimerase [EC:5.1.3.2] | | | | | |  | |  |
|  | ko:K07508 ACAA2; acetyl-CoA acyltransferase 2 [EC:2.3.1.16] | | | | | |  | |  |
|  | ko:K08048 ADCY8; adenylate cyclase 8 [EC:4.6.1.1] | | | | |  |  | |  |
|  | ko:K10247 ELOVL1; elongation of very long chain fatty acids protein 1 [EC:2.3.1.199] | | | | | | | | |
| ko01110 Biosynthesis of secondary metabolites (8) | | | | | |  |  | |  |
|  | ko:K00010 iolG; myo-inositol 2-dehydrogenase / D-chiro-inositol 1-dehydrogenase [EC:1.1.1.18 1.1.1.369] | | | | | | | | |
|  | ko:K00134 GAPDH; glyceraldehyde 3-phosphate dehydrogenase [EC:1.2.1.12] | | | | | | | | |
|  | ko:K00281 GLDC; glycine dehydrogenase [EC:1.4.4.2] | | | | | |  | |  |
|  | ko:K00430 E1.11.1.7; peroxidase [EC:1.11.1.7] | | | | |  |  | |  |
|  | ko:K00948 PRPS; ribose-phosphate pyrophosphokinase [EC:2.7.6.1] | | | | | |  | |  |
|  | ko:K07508 ACAA2; acetyl-CoA acyltransferase 2 [EC:2.3.1.16] | | | | | |  | |  |
|  | ko:K10247 ELOVL1; elongation of very long chain fatty acids protein 1 [EC:2.3.1.199] | | | | | | | | |
|  | ko:K15890 FOHSDR; NADP+-dependent farnesol dehydrogenase [EC:1.1.1.216] | | | | | | | | |
| ko01200 Carbon metabolism (3) | | | |  | |  |  | |  |
|  | ko:K00134 GAPDH; glyceraldehyde 3-phosphate dehydrogenase [EC:1.2.1.12] | | | | | | | | |
|  | ko:K00281 GLDC; glycine dehydrogenase [EC:1.4.4.2] | | | | | |  | |  |
|  | ko:K00948 PRPS; ribose-phosphate pyrophosphokinase [EC:2.7.6.1] | | | | | |  | |  |
| ko01120 Microbial metabolism in diverse environments (3) | | | | | | |  | |  |
|  | ko:K00010 iolG; myo-inositol 2-dehydrogenase / D-chiro-inositol 1-dehydrogenase [EC:1.1.1.18 1.1.1.369] | | | | | | | | |
|  | ko:K00134 GAPDH; glyceraldehyde 3-phosphate dehydrogenase [EC:1.2.1.12] | | | | | | | | |
|  | ko:K00948 PRPS; ribose-phosphate pyrophosphokinase [EC:2.7.6.1] | | | | | |  | |  |
| ko05010 Alzheimer disease (3) | | | |  | |  |  | |  |
|  | ko:K00134 GAPDH; glyceraldehyde 3-phosphate dehydrogenase [EC:1.2.1.12] | | | | | | | | |
|  | ko:K02183 CALM; calmodulin | | |  | |  |  | |  |
|  | ko:K07375 TUBB; tubulin beta | | |  | |  |  | |  |
| ko05130 Pathogenic Escherichia coli infection (3) | | | | | |  |  | |  |
|  | ko:K00134 GAPDH; glyceraldehyde 3-phosphate dehydrogenase [EC:1.2.1.12] | | | | | | | | |
|  | ko:K05692 ACTB_G1; actin beta/gamma 1 | | | | |  |  | |  |
|  | ko:K07375 TUBB; tubulin beta | | |  | |  |  | |  |
| ko00260 Glycine, serine and threonine metabolism (3) | | | | | |  |  | |  |
|  | ko:K00108 betA; choline dehydrogenase [EC:1.1.99.1] | | | | | |  | |  |
|  | ko:K00281 GLDC; glycine dehydrogenase [EC:1.4.4.2] | | | | | |  | |  |
|  | ko:K00552 GNMT; glycine N-methyltransferase [EC:2.1.1.20] | | | | | |  | |  |
| ko04921 Oxytocin signaling pathway (3) | | | | | |  |  | |  |
|  | ko:K02183 CALM; calmodulin | | |  | |  |  | |  |
|  | ko:K05692 ACTB_G1; actin beta/gamma 1 | | | | |  |  | |  |
|  | ko:K08048 ADCY8; adenylate cyclase 8 [EC:4.6.1.1] | | | | |  |  | |  |
| ko05132 Salmonella infection (3) | | | |  | |  |  | |  |
|  | ko:K00134 GAPDH; glyceraldehyde 3-phosphate dehydrogenase [EC:1.2.1.12] | | | | | | | | |
|  | ko:K05692 ACTB_G1; actin beta/gamma 1 | | | | |  |  | |  |
|  | ko:K07375 TUBB; tubulin beta | | |  | |  |  | |  |
| ko04971 Gastric acid secretion (3) | | | |  | |  |  | |  |
|  | ko:K02183 CALM; calmodulin | | |  | |  |  | |  |
|  | ko:K05692 ACTB_G1; actin beta/gamma 1 | | | | |  |  | |  |
|  | ko:K08048 ADCY8; adenylate cyclase 8 [EC:4.6.1.1] | | | | |  |  | |  |
| ko04015 Rap1 signaling pathway (3) | | | |  | |  |  | |  |
|  | ko:K02183 CALM; calmodulin | | |  | |  |  | |  |
|  | ko:K05692 ACTB_G1; actin beta/gamma 1 | | | | |  |  | |  |
|  | ko:K08048 ADCY8; adenylate cyclase 8 [EC:4.6.1.1] | | | | |  |  | |  |
| ko04145 Phagosome (2) | | | |  | |  |  | |  |
|  | ko:K05692 ACTB_G1; actin beta/gamma 1 | | | | |  |  | |  |
|  | ko:K07375 TUBB; tubulin beta | | |  | |  |  | |  |
| ko05200 Pathways in cancer (2) | | | |  | |  |  | |  |
|  | ko:K02183 CALM; calmodulin | | |  | |  |  | |  |
|  | ko:K08048 ADCY8; adenylate cyclase 8 [EC:4.6.1.1] | | | | |  |  | |  |
| ko04020 Calcium signaling pathway (2) | | | |  | |  |  | |  |
|  | ko:K02183 CALM; calmodulin | | |  | |  |  | |  |
|  | ko:K08048 ADCY8; adenylate cyclase 8 [EC:4.6.1.1] | | | | |  |  | |  |
| ko04916 Melanogenesis (2) | | | |  | |  |  | |  |
|  | ko:K02183 CALM; calmodulin | | |  | |  |  | |  |
|  | ko:K08048 ADCY8; adenylate cyclase 8 [EC:4.6.1.1] | | | | |  |  | |  |
| ko04974 Protein digestion and absorption (2) | | | | | |  |  | |  |
|  | ko:K01312 PRSS1_2_3; trypsin [EC:3.4.21.4] | | | | |  |  | |  |
|  | ko:K13868 SLC7A9_15; solute carrier family 7 (L-type amino acid transporter), member 9/15 | | | | | | | | |
| ko04261 Adrenergic signaling in cardiomyocytes (2) | | | | | |  |  | |  |
|  | ko:K02183 CALM; calmodulin | | |  | |  |  | |  |
|  | ko:K08048 ADCY8; adenylate cyclase 8 [EC:4.6.1.1] | | | | |  |  | |  |
| ko04611 Platelet activation (2) | | | |  | |  |  | |  |
|  | ko:K05692 ACTB_G1; actin beta/gamma 1 | | | | |  |  | |  |
|  | ko:K08048 ADCY8; adenylate cyclase 8 [EC:4.6.1.1] | | | | |  |  | |  |
| ko05022 Pathways of neurodegeneration - multiple diseases (2) | | | | | | |  | |  |
|  | ko:K02183 CALM; calmodulin | | |  | |  |  | |  |
|  | ko:K07375 TUBB; tubulin beta | | |  | |  |  | |  |
| ko04912 GnRH signaling pathway (2) | | | |  | |  |  | |  |
|  | ko:K02183 CALM; calmodulin | | |  | |  |  | |  |
|  | ko:K08048 ADCY8; adenylate cyclase 8 [EC:4.6.1.1] | | | | |  |  | |  |
| ko04371 Apelin signaling pathway (2) | | | |  | |  |  | |  |
|  | ko:K02183 CALM; calmodulin | | |  | |  |  | |  |
|  | ko:K08048 ADCY8; adenylate cyclase 8 [EC:4.6.1.1] | | | | |  |  | |  |
| ko01212 Fatty acid metabolism (2) | | | |  | |  |  | |  |
|  | ko:K07508 ACAA2; acetyl-CoA acyltransferase 2 [EC:2.3.1.16] | | | | | |  | |  |
|  | ko:K10247 ELOVL1; elongation of very long chain fatty acids protein 1 [EC:2.3.1.199] | | | | | | | | |
| ko05418 Fluid shear stress and atherosclerosis (2) | | | | | |  |  | |  |
|  | ko:K02183 CALM; calmodulin | | |  | |  |  | |  |
|  | ko:K05692 ACTB_G1; actin beta/gamma 1 | | | | |  |  | |  |
| ko01230 Biosynthesis of amino acids (2) | | | | | |  |  | |  |
|  | ko:K00134 GAPDH; glyceraldehyde 3-phosphate dehydrogenase [EC:1.2.1.12] | | | | | | | | |
|  | ko:K00948 PRPS; ribose-phosphate pyrophosphokinase [EC:2.7.6.1] | | | | | |  | |  |
| ko04714 Thermogenesis (2) | | | |  | |  |  | |  |
|  | ko:K05692 ACTB_G1; actin beta/gamma 1 | | | | |  |  | |  |
|  | ko:K08048 ADCY8; adenylate cyclase 8 [EC:4.6.1.1] | | | | |  |  | |  |
| ko04114 Oocyte meiosis (2) | | | |  | |  |  | |  |
|  | ko:K02183 CALM; calmodulin | | |  | |  |  | |  |
|  | ko:K08048 ADCY8; adenylate cyclase 8 [EC:4.6.1.1] | | | | |  |  | |  |
| ko04972 Pancreatic secretion (2) | | | |  | |  |  | |  |
|  | ko:K01312 PRSS1_2_3; trypsin [EC:3.4.21.4] | | | | |  |  | |  |
|  | ko:K08048 ADCY8; adenylate cyclase 8 [EC:4.6.1.1] | | | | |  |  | |  |
| ko05012 Parkinson disease (2) | | | |  | |  |  | |  |
|  | ko:K02183 CALM; calmodulin | | |  | |  |  | |  |
|  | ko:K07375 TUBB; tubulin beta | | |  | |  |  | |  |
| ko00230 Purine metabolism (2) | | | |  | |  |  | |  |
|  | ko:K00948 PRPS; ribose-phosphate pyrophosphokinase [EC:2.7.6.1] | | | | | |  | |  |
|  | ko:K08048 ADCY8; adenylate cyclase 8 [EC:4.6.1.1] | | | | |  |  | |  |
| ko05414 Dilated cardiomyopathy (2) | | | |  | |  |  | |  |
|  | ko:K05692 ACTB_G1; actin beta/gamma 1 | | | | |  |  | |  |
|  | ko:K08048 ADCY8; adenylate cyclase 8 [EC:4.6.1.1] | | | | |  |  | |  |
| ko04914 Progesterone-mediated oocyte maturation (2) | | | | | |  |  | |  |
|  | ko:K03364 CDH1; cell division cycle 20-like protein 1, cofactor of APC complex | | | | | | | | |
|  | ko:K08048 ADCY8; adenylate cyclase 8 [EC:4.6.1.1] | | | | |  |  | |  |
| ko00062 Fatty acid elongation (2) | | | |  | |  |  | |  |
|  | ko:K07508 ACAA2; acetyl-CoA acyltransferase 2 [EC:2.3.1.16] | | | | | |  | |  |
|  | ko:K10247 ELOVL1; elongation of very long chain fatty acids protein 1 [EC:2.3.1.199] | | | | | | | | |
| ko04970 Salivary secretion (2) | | | |  | |  |  | |  |
|  | ko:K02183 CALM; calmodulin | | |  | |  |  | |  |
|  | ko:K08048 ADCY8; adenylate cyclase 8 [EC:4.6.1.1] | | | | |  |  | |  |
| ko05163 Human cytomegalovirus infection (2) | | | | | |  |  | |  |
|  | ko:K02183 CALM; calmodulin | | |  | |  |  | |  |
|  | ko:K08048 ADCY8; adenylate cyclase 8 [EC:4.6.1.1] | | | | |  |  | |  |
| ko04745 Phototransduction - fly (2) | | | |  | |  |  | |  |
|  | ko:K02183 CALM; calmodulin | | |  | |  |  | |  |
|  | ko:K05692 ACTB_G1; actin beta/gamma 1 | | | | |  |  | |  |
| ko04270 Vascular smooth muscle contraction (2) | | | | | |  |  | |  |
|  | ko:K02183 CALM; calmodulin | | |  | |  |  | |  |
|  | ko:K08048 ADCY8; adenylate cyclase 8 [EC:4.6.1.1] | | | | |  |  | |  |
| ko04022 cGMP-PKG signaling pathway (2) | | | | | |  |  | |  |
|  | ko:K02183 CALM; calmodulin | | |  | |  |  | |  |
|  | ko:K08048 ADCY8; adenylate cyclase 8 [EC:4.6.1.1] | | | | |  |  | |  |
| ko00981 Insect hormone biosynthesis (2) | | | | | |  |  | |  |
|  | ko:K10718 JHAMT; juvenile hormone-III synthase [EC:2.1.1.325] | | | | | |  | |  |
|  | ko:K15890 FOHSDR; NADP+-dependent farnesol dehydrogenase [EC:1.1.1.216] | | | | | | | | |
| ko04713 Circadian entrainment (2) | | | |  | |  |  | |  |
|  | ko:K02183 CALM; calmodulin | | |  | |  |  | |  |
|  | ko:K08048 ADCY8; adenylate cyclase 8 [EC:4.6.1.1] | | | | |  |  | |  |
| ko05014 Amyotrophic lateral sclerosis (2) | | | | | |  |  | |  |
|  | ko:K05692 ACTB_G1; actin beta/gamma 1 | | | | |  |  | |  |
|  | ko:K07375 TUBB; tubulin beta | | |  | |  |  | |  |
| ko04925 Aldosterone synthesis and secretion (2) | | | | | |  |  | |  |
|  | ko:K02183 CALM; calmodulin | | |  | |  |  | |  |
|  | ko:K08048 ADCY8; adenylate cyclase 8 [EC:4.6.1.1] | | | | |  |  | |  |
| ko04750 Inflammatory mediator regulation of TRP channels (2) | | | | | | |  | |  |
|  | ko:K02183 CALM; calmodulin | | |  | |  |  | |  |
|  | ko:K08048 ADCY8; adenylate cyclase 8 [EC:4.6.1.1] | | | | |  |  | |  |
| ko05164 Influenza A (2) | | | |  | |  |  | |  |
|  | ko:K01312 PRSS1_2_3; trypsin [EC:3.4.21.4] | | | | |  |  | |  |
|  | ko:K05692 ACTB_G1; actin beta/gamma 1 | | | | |  |  | |  |
| ko04915 Estrogen signaling pathway (2) | | | | | |  |  | |  |
|  | ko:K02183 CALM; calmodulin | | |  | |  |  | |  |
|  | ko:K08048 ADCY8; adenylate cyclase 8 [EC:4.6.1.1] | | | | |  |  | |  |
| ko04540 Gap junction (2) | | | |  | |  |  | |  |
|  | ko:K07375 TUBB; tubulin beta | | |  | |  |  | |  |
|  | ko:K08048 ADCY8; adenylate cyclase 8 [EC:4.6.1.1] | | | | |  |  | |  |
| ko04024 cAMP signaling pathway (2) | | | |  | |  |  | |  |
|  | ko:K02183 CALM; calmodulin | | |  | |  |  | |  |
|  | ko:K08048 ADCY8; adenylate cyclase 8 [EC:4.6.1.1] | | | | |  |  | |  |
| ko04720 Long-term potentiation (2) | | | |  | |  |  | |  |
|  | ko:K02183 CALM; calmodulin | | |  | |  |  | |  |
|  | ko:K08048 ADCY8; adenylate cyclase 8 [EC:4.6.1.1] | | | | |  |  | |  |
| ko00562 Inositol phosphate metabolism (1) | | | | | |  |  | |  |
|  | ko:K00010 iolG; myo-inositol 2-dehydrogenase / D-chiro-inositol 1-dehydrogenase [EC:1.1.1.18 1.1.1.369] | | | | | | | | |
| ko04510 Focal adhesion (1) | | | |  | |  |  | |  |
|  | ko:K05692 ACTB_G1; actin beta/gamma 1 | | | | |  |  | |  |
| ko04613 Neutrophil extracellular trap formation (1) | | | | | |  |  | |  |
|  | ko:K05692 ACTB_G1; actin beta/gamma 1 | | | | |  |  | |  |
| ko04080 Neuroactive ligand-receptor interaction (1) | | | | | |  |  | |  |
|  | ko:K01312 PRSS1_2_3; trypsin [EC:3.4.21.4] | | | | |  |  | |  |
| ko00900 Terpenoid backbone biosynthesis (1) | | | | | |  |  | |  |
|  | ko:K15890 FOHSDR; NADP+-dependent farnesol dehydrogenase [EC:1.1.1.216] | | | | | | | | |
| ko00940 Phenylpropanoid biosynthesis (1) | | | | | |  |  | |  |
|  | ko:K00430 E1.11.1.7; peroxidase [EC:1.11.1.7] | | | | |  |  | |  |
| ko04924 Renin secretion (1) | | | |  | |  |  | |  |
|  | ko:K02183 CALM; calmodulin | | |  | |  |  | |  |
| ko04210 Apoptosis (1) | | | |  | |  |  | |  |
|  | ko:K05692 ACTB_G1; actin beta/gamma 1 | | | | |  |  | |  |
| ko04727 GABAergic synapse (1) | | | |  | |  |  | |  |
|  | ko:K08048 ADCY8; adenylate cyclase 8 [EC:4.6.1.1] | | | | |  |  | |  |
| ko05415 Diabetic cardiomyopathy (1) | | | |  | |  |  | |  |
|  | ko:K00134 GAPDH; glyceraldehyde 3-phosphate dehydrogenase [EC:1.2.1.12] | | | | | | | | |
| ko00541 O-Antigen nucleotide sugar biosynthesis (1) | | | | | |  |  | |  |
|  | ko:K01784 galE; UDP-glucose 4-epimerase [EC:5.1.3.2] | | | | | |  | |  |
| ko04110 Cell cycle (1) | | | |  | |  |  | |  |
|  | ko:K03364 CDH1; cell division cycle 20-like protein 1, cofactor of APC complex | | | | | | | | |
| ko04978 Mineral absorption (1) | | | |  | |  |  | |  |
|  | ko:K14686 SLC31A1; solute carrier family 31 (copper transporter), member 1 | | | | | | | | |
| ko05110 Vibrio cholerae infection (1) | | | |  | |  |  | |  |
|  | ko:K05692 ACTB_G1; actin beta/gamma 1 | | | | |  |  | |  |
| ko04926 Relaxin signaling pathway (1) | | | |  | |  |  | |  |
|  | ko:K08048 ADCY8; adenylate cyclase 8 [EC:4.6.1.1] | | | | |  |  | |  |
| ko00030 Pentose phosphate pathway (1) | | | | | |  |  | |  |
|  | ko:K00948 PRPS; ribose-phosphate pyrophosphokinase [EC:2.7.6.1] | | | | | |  | |  |
| ko03320 PPAR signaling pathway (1) | | | |  | |  |  | |  |
|  | ko:K08752 FABP3; fatty acid-binding protein 3, muscle and heart | | | | | |  | |  |
| ko01040 Biosynthesis of unsaturated fatty acids (1) | | | | | |  |  | |  |
|  | ko:K10247 ELOVL1; elongation of very long chain fatty acids protein 1 [EC:2.3.1.199] | | | | | | | | |
| ko04723 Retrograde endocannabinoid signaling (1) | | | | | |  |  | |  |
|  | ko:K08048 ADCY8; adenylate cyclase 8 [EC:4.6.1.1] | | | | |  |  | |  |
| ko04530 Tight junction (1) | | | |  | |  |  | |  |
|  | ko:K05692 ACTB_G1; actin beta/gamma 1 | | | | |  |  | |  |
| ko04390 Hippo signaling pathway (1) | | | |  | |  |  | |  |
|  | ko:K05692 ACTB_G1; actin beta/gamma 1 | | | | |  |  | |  |
| ko04625 C-type lectin receptor signaling pathway (1) | | | | | |  |  | |  |
|  | ko:K02183 CALM; calmodulin | | |  | |  |  | |  |
| ko04728 Dopaminergic synapse (1) | | | |  | |  |  | |  |
|  | ko:K02183 CALM; calmodulin | | |  | |  |  | |  |
| ko04742 Taste transduction (1) | | | |  | |  |  | |  |
|  | ko:K08048 ADCY8; adenylate cyclase 8 [EC:4.6.1.1] | | | | |  |  | |  |
| ko05135 Yersinia infection (1) | | | |  | |  |  | |  |
|  | ko:K05692 ACTB_G1; actin beta/gamma 1 | | | | |  |  | |  |
| ko00052 Galactose metabolism (1) | | | |  | |  |  | |  |
|  | ko:K01784 galE; UDP-glucose 4-epimerase [EC:5.1.3.2] | | | | | |  | |  |
| ko04016 MAPK signaling pathway - plant (1) | | | | | |  |  | |  |
|  | ko:K02183 CALM; calmodulin | | |  | |  |  | |  |
| ko04213 Longevity regulating pathway - multiple species (1) | | | | | | |  | |  |
|  | ko:K08048 ADCY8; adenylate cyclase 8 [EC:4.6.1.1] | | | | |  |  | |  |
| ko04979 Cholesterol metabolism (1) | | | |  | |  |  | |  |
|  | ko:K01052 LIPA; lysosomal acid lipase/cholesteryl ester hydrolase [EC:3.1.1.13] | | | | | | | | |
| ko05412 Arrhythmogenic right ventricular cardiomyopathy (1) | | | | | | |  | |  |
|  | ko:K05692 ACTB_G1; actin beta/gamma 1 | | | | |  |  | |  |
| ko04911 Insulin secretion (1) | | | |  | |  |  | |  |
|  | ko:K08048 ADCY8; adenylate cyclase 8 [EC:4.6.1.1] | | | | |  |  | |  |
| ko04120 Ubiquitin mediated proteolysis (1) | | | | | |  |  | |  |
|  | ko:K03364 CDH1; cell division cycle 20-like protein 1, cofactor of APC complex | | | | | | | | |
| ko05416 Viral myocarditis (1) | | | |  | |  |  | |  |
|  | ko:K05692 ACTB_G1; actin beta/gamma 1 | | | | |  |  | |  |
| ko00520 Amino sugar and nucleotide sugar metabolism (1) | | | | | | |  | |  |
|  | ko:K01784 galE; UDP-glucose 4-epimerase [EC:5.1.3.2] | | | | | |  | |  |
| ko01524 Platinum drug resistance (1) | | | |  | |  |  | |  |
|  | ko:K14686 SLC31A1; solute carrier family 31 (copper transporter), member 1 | | | | | | | | |
| ko04211 Longevity regulating pathway (1) | | | | | |  |  | |  |
|  | ko:K08048 ADCY8; adenylate cyclase 8 [EC:4.6.1.1] | | | | |  |  | |  |
| ko01522 Endocrine resistance (1) | | | |  | |  |  | |  |
|  | ko:K08048 ADCY8; adenylate cyclase 8 [EC:4.6.1.1] | | | |  | | |  | |
| ko05214 Glioma (1) | | |  | |  | | |  | |
|  | ko:K02183 CALM; calmodulin |  | | |  | | |  | |
| ko04910 Insulin signaling pathway (1) | | | |  | |  |  | |  |
|  | ko:K02183 CALM; calmodulin | | |  | |  |  | |  |
| ko04725 Cholinergic synapse (1) | | | |  | |  |  | |  |
|  | ko:K08048 ADCY8; adenylate cyclase 8 [EC:4.6.1.1] | | | | |  |  | |  |
| ko04934 Cushing syndrome (1) | | | |  | |  |  | |  |
|  | ko:K08048 ADCY8; adenylate cyclase 8 [EC:4.6.1.1] | | | | |  |  | |  |
| ko04722 Neurotrophin signaling pathway (1) | | | | | |  |  | |  |
|  | ko:K02183 CALM; calmodulin | | |  | |  |  | |  |
| ko05417 Lipid and atherosclerosis (1) | | | |  | |  |  | |  |
|  | ko:K02183 CALM; calmodulin | | |  | |  |  | |  |
| ko00071 Fatty acid degradation (1) | | | |  | |  |  | |  |
|  | ko:K07508 ACAA2; acetyl-CoA acyltransferase 2 [EC:2.3.1.16] | | | | | |  | |  |
| ko05170 Human immunodeficiency virus 1 infection (1) | | | | | |  |  | |  |
|  | ko:K02183 CALM; calmodulin | | |  | |  |  | |  |
| ko05167 Kaposi sarcoma-associated herpesvirus infection (1) | | | | | | |  | |  |
|  | ko:K02183 CALM; calmodulin | | |  | |  |  | |  |
| ko00910 Nitrogen metabolism (1) | | | |  | |  |  | |  |
|  | ko:K01672 CA; carbonic anhydrase [EC:4.2.1.1] | | | | |  |  | |  |
| ko03013 RNA transport (1) | | | |  | |  |  | |  |
|  | ko:K14525 RPP25; ribonucleases P/MRP protein subunit RPP25 [EC:3.1.26.5] | | | | | | | | |
| ko00630 Glyoxylate and dicarboxylate metabolism (1) | | | | | |  |  | |  |
|  | ko:K00281 GLDC; glycine dehydrogenase [EC:1.4.4.2] | | | | | |  | |  |
| ko04724 Glutamatergic synapse (1) | | | |  | |  |  | |  |
|  | ko:K08048 ADCY8; adenylate cyclase 8 [EC:4.6.1.1] | | | | |  |  | |  |
| ko04391 Hippo signaling pathway - fly (1) | | | | | |  |  | |  |
|  | ko:K05692 ACTB_G1; actin beta/gamma 1 | | | | |  |  | |  |
| ko00380 Tryptophan metabolism (1) | | | |  | |  |  | |  |
|  | ko:K00453 TDO2; tryptophan 2,3-dioxygenase [EC:1.13.11.11] | | | | | |  | |  |
| ko04919 Thyroid hormone signaling pathway (1) | | | | | |  |  | |  |
|  | ko:K05692 ACTB_G1; actin beta/gamma 1 | | | | |  |  | |  |
| ko04976 Bile secretion (1) | | | |  | |  |  | |  |
|  | ko:K08048 ADCY8; adenylate cyclase 8 [EC:4.6.1.1] | | | | |  |  | |  |
| ko05100 Bacterial invasion of epithelial cells (1) | | | | | |  |  | |  |
|  | ko:K05692 ACTB_G1; actin beta/gamma 1 | | | | |  |  | |  |
| ko04935 Growth hormone synthesis, secretion and action (1) | | | | | | |  | |  |
|  | ko:K08048 ADCY8; adenylate cyclase 8 [EC:4.6.1.1] | | | | |  |  | |  |
| ko05133 Pertussis (1) | | | |  | |  |  | |  |
|  | ko:K02183 CALM; calmodulin | | |  | |  |  | |  |
| ko04626 Plant-pathogen interaction (1) | | | | | |  |  | |  |
|  | ko:K02183 CALM; calmodulin | | |  | |  |  | |  |
| ko00010 Glycolysis / Gluconeogenesis (1) | | | | | |  |  | |  |
|  | ko:K00134 GAPDH; glyceraldehyde 3-phosphate dehydrogenase [EC:1.2.1.12] | | | | | | | | |
| ko04146 Peroxisome (1) | | | |  | |  |  | |  |
|  | ko:K13348 MPV17; protein Mpv17 | | | | |  |  | |  |
| ko04066 HIF-1 signaling pathway (1) | | | |  | |  |  | |  |
|  | ko:K00134 GAPDH; glyceraldehyde 3-phosphate dehydrogenase [EC:1.2.1.12] | | | | | | | | |
| ko00100 Steroid biosynthesis (1) | | | |  | |  |  | |  |
|  | ko:K01052 LIPA; lysosomal acid lipase/cholesteryl ester hydrolase [EC:3.1.1.13] | | | | | | | | |
| ko04927 Cortisol synthesis and secretion (1) | | | | | |  |  | |  |
|  | ko:K08048 ADCY8; adenylate cyclase 8 [EC:4.6.1.1] | | | | |  |  | |  |
| ko05034 Alcoholism (1) | | | |  | |  |  | |  |
|  | ko:K02183 CALM; calmodulin | | |  | |  |  | |  |
| ko00521 Streptomycin biosynthesis (1) | | | | | |  |  | |  |
|  | ko:K00010 iolG; myo-inositol 2-dehydrogenase / D-chiro-inositol 1-dehydrogenase [EC:1.1.1.18 1.1.1.369] | | | | | | | | |
| ko04062 Chemokine signaling pathway (1) | | | | | |  |  | |  |
|  | ko:K08048 ADCY8; adenylate cyclase 8 [EC:4.6.1.1] | | | | |  |  | |  |
| ko05016 Huntington disease (1) | | | |  | |  |  | |  |
|  | ko:K07375 TUBB; tubulin beta | | |  | |  |  | |  |
| ko05410 Hypertrophic cardiomyopathy (1) | | | | | |  |  | |  |
|  | ko:K05692 ACTB_G1; actin beta/gamma 1 | | | | |  |  | |  |
| ko01240 Biosynthesis of cofactors (1) | | | |  | |  |  | |  |
|  | ko:K00453 TDO2; tryptophan 2,3-dioxygenase [EC:1.13.11.11] | | | | | |  | |  |
| ko04913 Ovarian steroidogenesis (1) | | | |  | |  |  | |  |
|  | ko:K08048 ADCY8; adenylate cyclase 8 [EC:4.6.1.1] | | | | |  |  | |  |
| ko04014 Ras signaling pathway (1) | | | |  | |  |  | |  |
|  | ko:K02183 CALM; calmodulin | | |  | |  |  | |  |
| ko05131 Shigellosis (1) | | | |  | |  |  | |  |
|  | ko:K05692 ACTB_G1; actin beta/gamma 1 | | | | |  |  | |  |
| ko00532 Glycosaminoglycan biosynthesis - chondroitin sulfate / dermatan sulfate (1) | | | | | | | | | |
|  | ko:K03193 UST; dermatan/chondrotin sulfate uronyl 2-O-sulfotransferase UST [EC:2.8.2.-] | | | | | | | | |
| ko05225 Hepatocellular carcinoma (1) | | | |  | |  |  | |  |
|  | ko:K05692 ACTB_G1; actin beta/gamma 1 | | | | |  |  | |  |
| ko05152 Tuberculosis (1) | | | |  | |  |  | |  |
|  | ko:K02183 CALM; calmodulin | | |  | |  |  | |  |
| ko00280 Valine, leucine and isoleucine degradation (1) | | | | | |  |  | |  |
|  | ko:K07508 ACAA2; acetyl-CoA acyltransferase 2 [EC:2.3.1.16] | | | | | |  | |  |
| ko03008 Ribosome biogenesis in eukaryotes (1) | | | | | |  |  | |  |
|  | ko:K14525 RPP25; ribonucleases P/MRP protein subunit RPP25 [EC:3.1.26.5] | | | | | | | | |
| ko04810 Regulation of actin cytoskeleton (1) | | | | | |  |  | |  |
|  | ko:K05692 ACTB_G1; actin beta/gamma 1 | | | | |  |  | |  |
| ko04918 Thyroid hormone synthesis (1) | | | | | |  |  | |  |
|  | ko:K08048 ADCY8; adenylate cyclase 8 [EC:4.6.1.1] | | | | |  |  | |  |
| ko04923 Regulation of lipolysis in adipocytes (1) | | | | | |  |  | |  |
|  | ko:K08048 ADCY8; adenylate cyclase 8 [EC:4.6.1.1] | | | | |  |  | |  |
| ko04670 Leukocyte transendothelial migration (1) | | | | | |  |  | |  |
|  | ko:K05692 ACTB_G1; actin beta/gamma 1 | | | | |  |  | |  |
| ko04740 Olfactory transduction (1) | | | |  | |  |  | |  |
|  | ko:K02183 CALM; calmodulin | | |  | |  |  | |  |
| ko00710 Carbon fixation in photosynthetic organisms (1) | | | | | | |  | |  |
|  | ko:K00134 GAPDH; glyceraldehyde 3-phosphate dehydrogenase [EC:1.2.1.12] | | | | | | | | |
| ko04111 Cell cycle - yeast (1) | | | |  | |  |  | |  |
|  | ko:K03364 CDH1; cell division cycle 20-like protein 1, cofactor of APC complex | | | | | | | | |
| ko04072 Phospholipase D signaling pathway (1) | | | | | |  |  | |  |
|  | ko:K08048 ADCY8; adenylate cyclase 8 [EC:4.6.1.1] | | | | |  |  | |  |
| ko05020 Prion disease (1) | | | |  | |  |  | |  |
|  | ko:K07375 TUBB; tubulin beta | | |  | |  |  | |  |
| ko04520 Adherens junction (1) | | | |  | |  |  | |  |
|  | ko:K05692 ACTB_G1; actin beta/gamma 1 | | | | |  |  | |  |
| ko04070 Phosphatidylinositol signaling system (1) | | | | | |  |  | |  |
|  | ko:K02183 CALM; calmodulin | | |  | |  |  | |  |
| ko04928 Parathyroid hormone synthesis, secretion and action (1) | | | | | | |  | |  |
|  | ko:K08048 ADCY8; adenylate cyclase 8 [EC:4.6.1.1] | | | | |  |  | |  |
| ko04142 Lysosome (1) | | | |  | |  |  | |  |
|  | ko:K01052 LIPA; lysosomal acid lipase/cholesteryl ester hydrolase [EC:3.1.1.13] | | | | | | | | |
| ko04922 Glucagon signaling pathway (1) | | | | | |  |  | |  |
|  | ko:K02183 CALM; calmodulin | | |  | |  |  | |  |
| ko05031 Amphetamine addiction (1) | | | |  | |  |  | |  |
|  | ko:K02183 CALM; calmodulin | | |  | |  |  | |  |
| ko05205 Proteoglycans in cancer (1) | | | |  | |  |  | |  |
|  | ko:K05692 ACTB_G1; actin beta/gamma 1 | | | | |  |  | |  |
| ko04218 Cellular senescence (1) | | | |  | |  |  | |  |
|  | ko:K02183 CALM; calmodulin | | |  | |  |  | |  |
| ko04744 Phototransduction (1) | | | |  | |  |  | |  |
|  | ko:K02183 CALM; calmodulin | | |  | |  |  | |  |
| ko05032 Morphine addiction (1) | | | |  | |  |  | |  |
|  | ko:K08048 ADCY8; adenylate cyclase 8 [EC:4.6.1.1] | | | | |  |  | |  |
| ko05166 Human T-cell leukemia virus 1 infection (1) | | | | | |  |  | |  |
|  | ko:K08048 ADCY8; adenylate cyclase 8 [EC:4.6.1.1] | | | | | |  | |  |

**Supplementary Table 10.** KEGG pathway hit results of downregulated genes

| ko01100 Metabolic pathways (14) | |
| --- | --- |
|  | ko:K00108 betA; choline dehydrogenase [EC:1.1.99.1] |
|  | ko:K00698 CHS1; chitin synthase [EC:2.4.1.16] |
|  | ko:K00699 UGT; glucuronosyltransferase [EC:2.4.1.17] |
|  | ko:K00736 MGAT2; alpha-1,6-mannosyl-glycoprotein beta-1,2-N-acetylglucosaminyltransferase [EC:2.4.1.143] |
|  | ko:K00827 AGXT2; alanine-glyoxylate transaminase / (R)-3-amino-2-methylpropionate-pyruvate transaminase [EC:2.6.1.44 2.6.1.40] |
|  | ko:K01179 E3.2.1.4; endoglucanase [EC:3.2.1.4] |
|  | ko:K01183 E3.2.1.14; chitinase [EC:3.2.1.14] |
|  | ko:K01769 E4.6.1.2; guanylate cyclase, other [EC:4.6.1.2] |
|  | ko:K03103 MINPP1; multiple inositol-polyphosphate phosphatase / 2,3-bisphosphoglycerate 3-phosphatase [EC:3.1.3.62 3.1.3.80] |
|  | ko:K05860 PLCE; phosphatidylinositol phospholipase C, epsilon [EC:3.1.4.11] |
|  | ko:K10250 ELOVL7; elongation of very long chain fatty acids protein 7 [EC:2.3.1.199] |
|  | ko:K12298 CEL; bile salt-stimulated lipase [EC:3.1.1.3 3.1.1.13] |
|  | ko:K14621 PLB1; phospholipase B1, membrane-associated [EC:3.1.1.4 3.1.1.5] |
|  | ko:K19970 NT5E; 5'-nucleotidase [EC:3.1.3.5] |
| ko01110 Biosynthesis of secondary metabolites (5) | |
|  | ko:K00699 UGT; glucuronosyltransferase [EC:2.4.1.17] |
|  | ko:K00827 AGXT2; alanine-glyoxylate transaminase / (R)-3-amino-2-methylpropionate-pyruvate transaminase [EC:2.6.1.44 2.6.1.40] |
|  | ko:K10250 ELOVL7; elongation of very long chain fatty acids protein 7 [EC:2.3.1.199] |
|  | ko:K14621 PLB1; phospholipase B1, membrane-associated [EC:3.1.1.4 3.1.1.5] |
|  | ko:K19970 NT5E; 5'-nucleotidase [EC:3.1.3.5] |
| ko04911 Insulin secretion (2) | |
|  | ko:K04944 KCNN3; potassium intermediate/small conductance calcium-activated channel subfamily N member 3 |
|  | ko:K15297 RIMS2; regulating synaptic membrane exocytosis protein 2 |
| ko00520 Amino sugar and nucleotide sugar metabolism (2) | |
|  | ko:K00698 CHS1; chitin synthase [EC:2.4.1.16] |
|  | ko:K01183 E3.2.1.14; chitinase [EC:3.2.1.14] |
| ko04977 Vitamin digestion and absorption (2) | |
|  | ko:K14616 CUBN; cubilin |
|  | ko:K14621 PLB1; phospholipase B1, membrane-associated [EC:3.1.1.4 3.1.1.5] |
| ko00564 Glycerophospholipid metabolism (2) | |
|  | ko:K01049 ACHE; acetylcholinesterase [EC:3.1.1.7] |
|  | ko:K14621 PLB1; phospholipase B1, membrane-associated [EC:3.1.1.4 3.1.1.5] |
| ko00260 Glycine, serine and threonine metabolism (2) | |
|  | ko:K00108 betA; choline dehydrogenase [EC:1.1.99.1] |
|  | ko:K00827 AGXT2; alanine-glyoxylate transaminase / (R)-3-amino-2-methylpropionate-pyruvate transaminase [EC:2.6.1.44 2.6.1.40] |
| ko04024 cAMP signaling pathway (2) | |
|  | ko:K05860 PLCE; phosphatidylinositol phospholipase C, epsilon [EC:3.1.4.11] |
|  | ko:K18435 SOX9; transcription factor SOX9 (SOX group E) |
| ko04080 Neuroactive ligand-receptor interaction (2) | |
|  | ko:K01312 PRSS1_2_3; trypsin [EC:3.4.21.4] |
|  | ko:K04157 HTR2; 5-hydroxytryptamine receptor 2 |
| ko04020 Calcium signaling pathway (2) | |
|  | ko:K04157 HTR2; 5-hydroxytryptamine receptor 2 |
|  | ko:K05860 PLCE; phosphatidylinositol phospholipase C, epsilon [EC:3.1.4.11] |
| ko04974 Protein digestion and absorption (2) | |
|  | ko:K01312 PRSS1_2_3; trypsin [EC:3.4.21.4] |
|  | ko:K06237 COL4A; collagen type IV alpha |
| ko04972 Pancreatic secretion (2) | |
|  | ko:K01312 PRSS1_2_3; trypsin [EC:3.4.21.4] |
|  | ko:K12298 CEL; bile salt-stimulated lipase [EC:3.1.1.3 3.1.1.13] |
| ko04933 AGE-RAGE signaling pathway in diabetic complications (2) | |
|  | ko:K05860 PLCE; phosphatidylinositol phospholipase C, epsilon [EC:3.1.4.11] |
|  | ko:K06237 COL4A; collagen type IV alpha |
| ko00230 Purine metabolism (2) | |
|  | ko:K01769 E4.6.1.2; guanylate cyclase, other [EC:4.6.1.2] |
|  | ko:K19970 NT5E; 5'-nucleotidase [EC:3.1.3.5] |
| ko00562 Inositol phosphate metabolism (2) | |
|  | ko:K03103 MINPP1; multiple inositol-polyphosphate phosphatase / 2,3-bisphosphoglycerate 3-phosphatase [EC:3.1.3.62 3.1.3.80] |
|  | ko:K05860 PLCE; phosphatidylinositol phospholipase C, epsilon [EC:3.1.4.11] |
| ko04550 Signaling pathways regulating pluripotency of stem cells (2) | |
|  | ko:K09370 ISL1; insulin gene enhancer protein ISL-1 |
|  | ko:K09378 ZFHX3; zinc finger homeobox protein 3 |
| ko04919 Thyroid hormone signaling pathway (1) | |
|  | ko:K05860 PLCE; phosphatidylinositol phospholipase C, epsilon [EC:3.1.4.11] |
| ko01040 Biosynthesis of unsaturated fatty acids (1) | |
|  | ko:K10250 ELOVL7; elongation of very long chain fatty acids protein 7 [EC:2.3.1.199] |
| ko04926 Relaxin signaling pathway (1) | |
|  | ko:K06237 COL4A; collagen type IV alpha |
| ko01212 Fatty acid metabolism (1) | |
|  | ko:K10250 ELOVL7; elongation of very long chain fatty acids protein 7 [EC:2.3.1.199] |
| ko00240 Pyrimidine metabolism (1) | |
|  | ko:K19970 NT5E; 5'-nucleotidase [EC:3.1.3.5] |
| ko00980 Metabolism of xenobiotics by cytochrome P450 (1) | |
|  | ko:K00699 UGT; glucuronosyltransferase [EC:2.4.1.17] |
| ko00270 Cysteine and methionine metabolism (1) | |
|  | ko:K00827 AGXT2; alanine-glyoxylate transaminase / (R)-3-amino-2-methylpropionate-pyruvate transaminase [EC:2.6.1.44 2.6.1.40] |
| ko04976 Bile secretion (1) | |
|  | ko:K00699 UGT; glucuronosyltransferase [EC:2.4.1.17] |
| ko00565 Ether lipid metabolism (1) | |
|  | ko:K14621 PLB1; phospholipase B1, membrane-associated [EC:3.1.1.4 3.1.1.5] |
| ko00250 Alanine, aspartate and glutamate metabolism (1) | |
|  | ko:K00827 AGXT2; alanine-glyoxylate transaminase / (R)-3-amino-2-methylpropionate-pyruvate transaminase [EC:2.6.1.44 2.6.1.40] |
| ko00500 Starch and sucrose metabolism (1) | |
|  | ko:K01179 E3.2.1.4; endoglucanase [EC:3.2.1.4] |
| ko00010 Glycolysis / Gluconeogenesis (1) | |
|  | ko:K03103 MINPP1; multiple inositol-polyphosphate phosphatase / 2,3-bisphosphoglycerate 3-phosphatase [EC:3.1.3.62 3.1.3.80] |
| ko00590 Arachidonic acid metabolism (1) | |
|  | ko:K14621 PLB1; phospholipase B1, membrane-associated [EC:3.1.1.4 3.1.1.5] |
| ko05131 Shigellosis (1) | |
|  | ko:K05860 PLCE; phosphatidylinositol phospholipase C, epsilon [EC:3.1.4.11] |
| ko04726 Serotonergic synapse (1) | |
|  | ko:K04157 HTR2; 5-hydroxytryptamine receptor 2 |
| ko00140 Steroid hormone biosynthesis (1) | |
|  | ko:K00699 UGT; glucuronosyltransferase [EC:2.4.1.17] |
| ko04961 Endocrine and other factor-regulated calcium reabsorption (1) | |
|  | ko:K04974 TRPV5; transient receptor potential cation channel subfamily V member 5 |
| ko05022 Pathways of neurodegeneration - multiple diseases (1) | |
|  | ko:K10408 DNAH; dynein heavy chain, axonemal |
| ko04070 Phosphatidylinositol signaling system (1) | |
|  | ko:K05860 PLCE; phosphatidylinositol phospholipase C, epsilon [EC:3.1.4.11] |
| ko00860 Porphyrin and chlorophyll metabolism (1) | |
|  | ko:K00699 UGT; glucuronosyltransferase [EC:2.4.1.17] |
| ko04151 PI3K-Akt signaling pathway (1) | |
|  | ko:K06237 COL4A; collagen type IV alpha |
| ko00561 Glycerolipid metabolism (1) | |
|  | ko:K12298 CEL; bile salt-stimulated lipase [EC:3.1.1.3 3.1.1.13] |
| ko05231 Choline metabolism in cancer (1) | |
|  | ko:K08202 SLC22A4_5; MFS transporter, OCT family, solute carrier family 22 (organic cation transporter), member 4/5 |
| ko04360 Axon guidance (1) | |
|  | ko:K06842 SEMA6; semaphorin 6 |
| ko04014 Ras signaling pathway (1) | |
|  | ko:K05860 PLCE; phosphatidylinositol phospholipase C, epsilon [EC:3.1.4.11] |
| ko00280 Valine, leucine and isoleucine degradation (1) | |
|  | ko:K00827 AGXT2; alanine-glyoxylate transaminase / (R)-3-amino-2-methylpropionate-pyruvate transaminase [EC:2.6.1.44 2.6.1.40] |
| ko04391 Hippo signaling pathway - fly (1) | |
|  | ko:K16669 FAT4; protocadherin Fat 4 |
| ko00983 Drug metabolism - other enzymes (1) | |
|  | ko:K00699 UGT; glucuronosyltransferase [EC:2.4.1.17] |
| ko04392 Hippo signaling pathway - multiple species (1) | |
|  | ko:K16669 FAT4; protocadherin Fat 4 |
| ko05204 Chemical carcinogenesis (1) | |
|  | ko:K00699 UGT; glucuronosyltransferase [EC:2.4.1.17] |
| ko00981 Insect hormone biosynthesis (1) | |
|  | ko:K14985 CYP18A1; 26-hydroxylase [EC:1.14.-.-] |
| ko02020 Two-component system (1) | |
|  | ko:K01179 E3.2.1.4; endoglucanase [EC:3.2.1.4] |
| ko00513 Various types of N-glycan biosynthesis (1) | |
|  | ko:K00736 MGAT2; alpha-1,6-mannosyl-glycoprotein beta-1,2-N-acetylglucosaminyltransferase [EC:2.4.1.143] |
| ko04015 Rap1 signaling pathway (1) | |
|  | ko:K05860 PLCE; phosphatidylinositol phospholipase C, epsilon [EC:3.1.4.11] |
| ko04540 Gap junction (1) | |
|  | ko:K04157 HTR2; 5-hydroxytryptamine receptor 2 |
| ko05222 Small cell lung cancer (1) | |
|  | ko:K06237 COL4A; collagen type IV alpha |
| ko05016 Huntington disease (1) | |
|  | ko:K10408 DNAH; dynein heavy chain, axonemal |
| ko04725 Cholinergic synapse (1) | |
|  | ko:K01049 ACHE; acetylcholinesterase [EC:3.1.1.7] |
| ko00760 Nicotinate and nicotinamide metabolism (1) | |
|  | ko:K19970 NT5E; 5'-nucleotidase [EC:3.1.3.5] |
| ko05146 Amoebiasis (1) | |
|  | ko:K06237 COL4A; collagen type IV alpha |
| ko01240 Biosynthesis of cofactors (1) | |
|  | ko:K00699 UGT; glucuronosyltransferase [EC:2.4.1.17] |
| ko04975 Fat digestion and absorption (1) | |
|  | ko:K12298 CEL; bile salt-stimulated lipase [EC:3.1.1.3 3.1.1.13] |
| ko05164 Influenza A (1) | |
|  | ko:K01312 PRSS1_2_3; trypsin [EC:3.4.21.4] |
| ko04721 Synaptic vesicle cycle (1) | |
|  | ko:K05038 SLC6A5_9; solute carrier family 6 (neurotransmitter transporter, glycine) member 5/9 |
| ko00062 Fatty acid elongation (1) | |
|  | ko:K10250 ELOVL7; elongation of very long chain fatty acids protein 7 [EC:2.3.1.199] |
| ko04510 Focal adhesion (1) | |
|  | ko:K06237 COL4A; collagen type IV alpha |
| ko00591 Linoleic acid metabolism (1) | |
|  | ko:K14621 PLB1; phospholipase B1, membrane-associated [EC:3.1.1.4 3.1.1.5] |
| ko05200 Pathways in cancer (1) | |
|  | ko:K06237 COL4A; collagen type IV alpha |
| ko04929 GnRH secretion (1) | |
|  | ko:K04944 KCNN3; potassium intermediate/small conductance calcium-activated channel subfamily N member 3 |
| ko04514 Cell adhesion molecules (1) | |
|  | ko:K07378 NLGN; neuroligin |
| ko00982 Drug metabolism - cytochrome P450 (1) | |
|  | ko:K00699 UGT; glucuronosyltransferase [EC:2.4.1.17] |
| ko05165 Human papillomavirus infection (1) | |
|  | ko:K06237 COL4A; collagen type IV alpha |
| ko04750 Inflammatory mediator regulation of TRP channels (1) | |
|  | ko:K04157 HTR2; 5-hydroxytryptamine receptor 2 |
| ko04928 Parathyroid hormone synthesis, secretion and action (1) | |
|  | ko:K04974 TRPV5; transient receptor potential cation channel subfamily V member 5 |
| ko00040 Pentose and glucuronate interconversions (1) | |
|  | ko:K00699 UGT; glucuronosyltransferase [EC:2.4.1.17] |
| ko00592 alpha-Linolenic acid metabolism (1) | |
|  | ko:K14621 PLB1; phospholipase B1, membrane-associated [EC:3.1.1.4 3.1.1.5] |
| ko05014 Amyotrophic lateral sclerosis (1) | |
|  | ko:K10408 DNAH; dynein heavy chain, axonemal |
| ko00510 N-Glycan biosynthesis (1) | |
|  | ko:K00736 MGAT2; alpha-1,6-mannosyl-glycoprotein beta-1,2-N-acetylglucosaminyltransferase [EC:2.4.1.143] |
| ko00053 Ascorbate and aldarate metabolism (1) | |
|  | ko:K00699 UGT; glucuronosyltransferase [EC:2.4.1.17] |
| ko04512 ECM-receptor interaction (1) | |
|  | ko:K06237 COL4A; collagen type IV alpha |
| ko00830 Retinol metabolism (1) | |
|  | ko:K00699 UGT; glucuronosyltransferase [EC:2.4.1.17] |
| ko00100 Steroid biosynthesis (1) | |
|  | ko:K12298 CEL; bile salt-stimulated lipase [EC:3.1.1.3 3.1.1.13] |
| ko05205 Proteoglycans in cancer (1) | |
|  | ko:K05860 PLCE; phosphatidylinositol phospholipase C, epsilon [EC:3.1.4.11] |


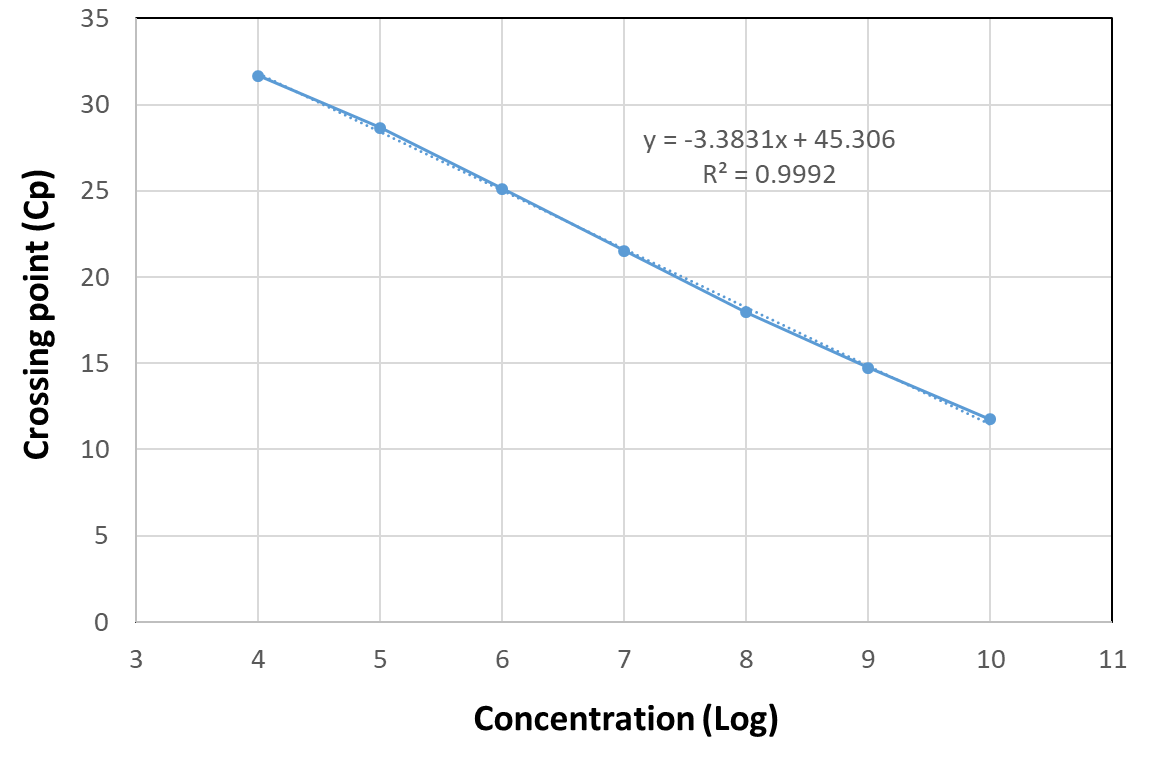


**Supplementary Figure 1.** Standard curve used to quantify DWV viral genome copies. The linear standard equation for DWV quantification was generated by plotting the crossing point (Cp) versus the log_10_ of the initial plasmid copy number.


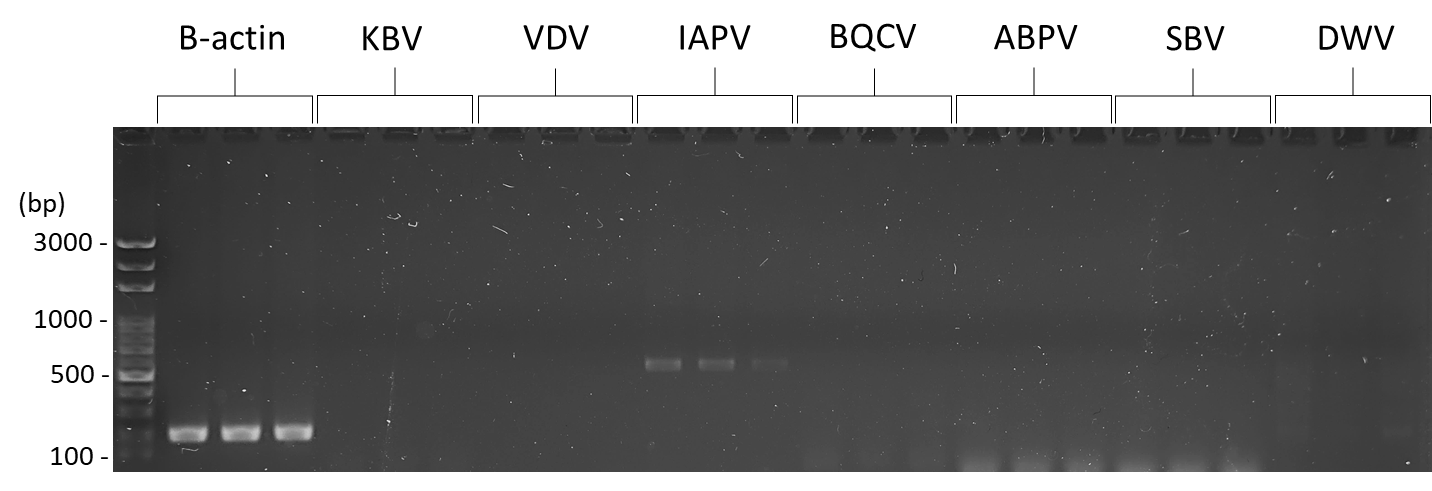


**Supplementary Figure 2.** The larvae of the honey bee colonies were screened for the prevalence of 7 viruses before DWV infection. Three replicates were performed in this screening.


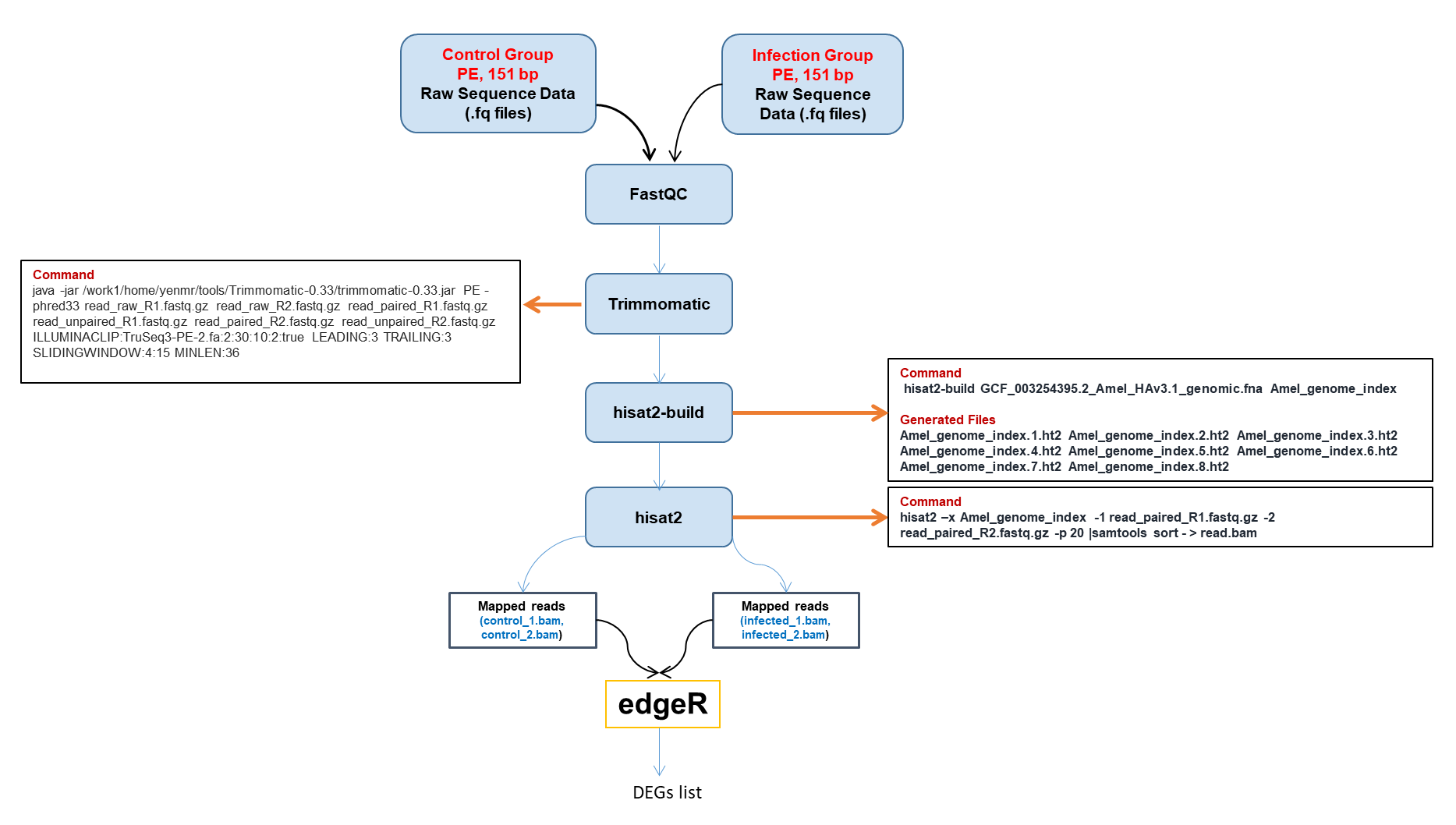


**Supplementary Figure 3.** Flowchart and codes of bioinformatics analyses.


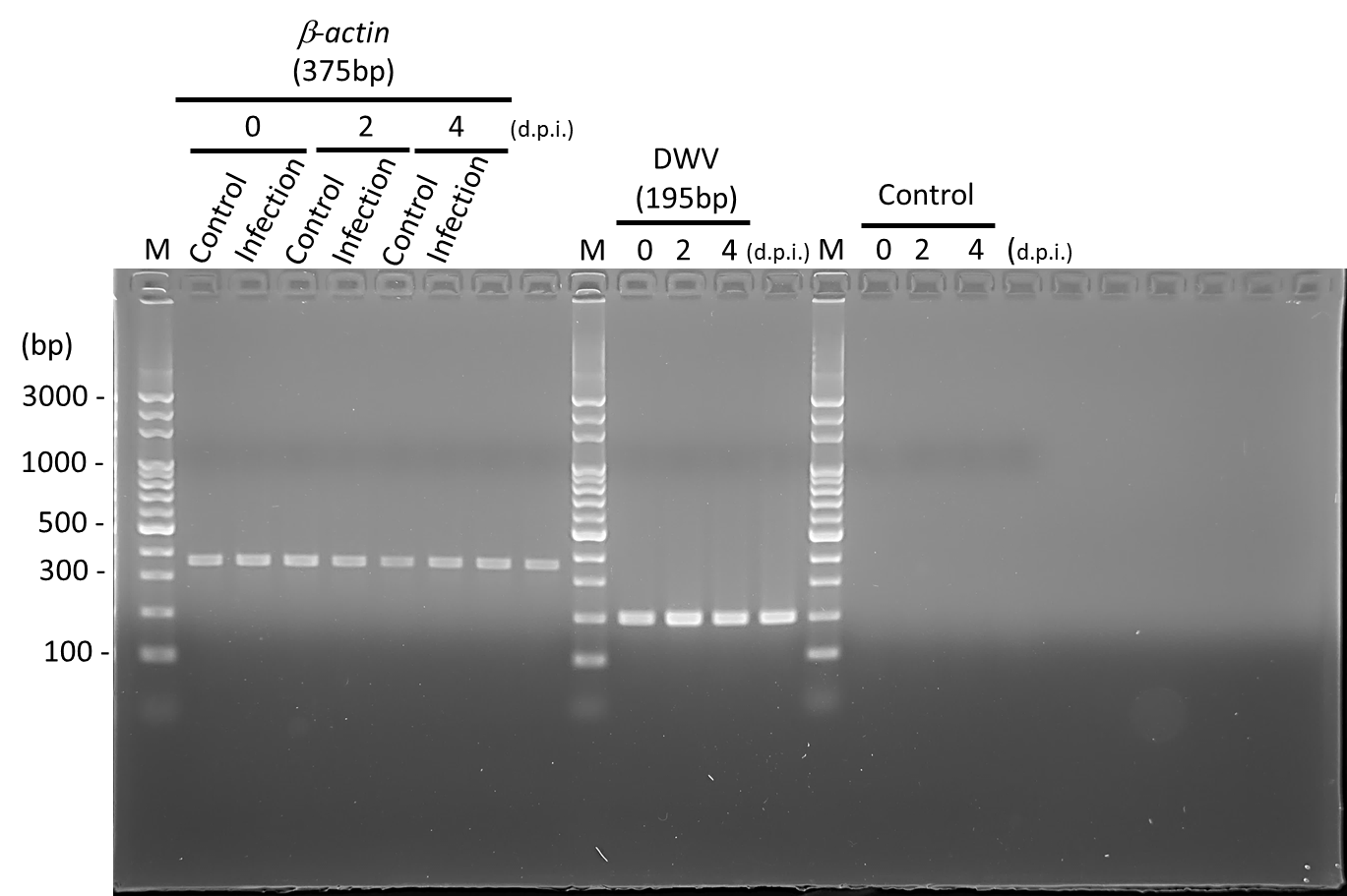


**Supplementary Figure 4.** The original DNA gel of the larval stage was cropped from different parts of this gel. This gel was re-edited in **Figure 2.**


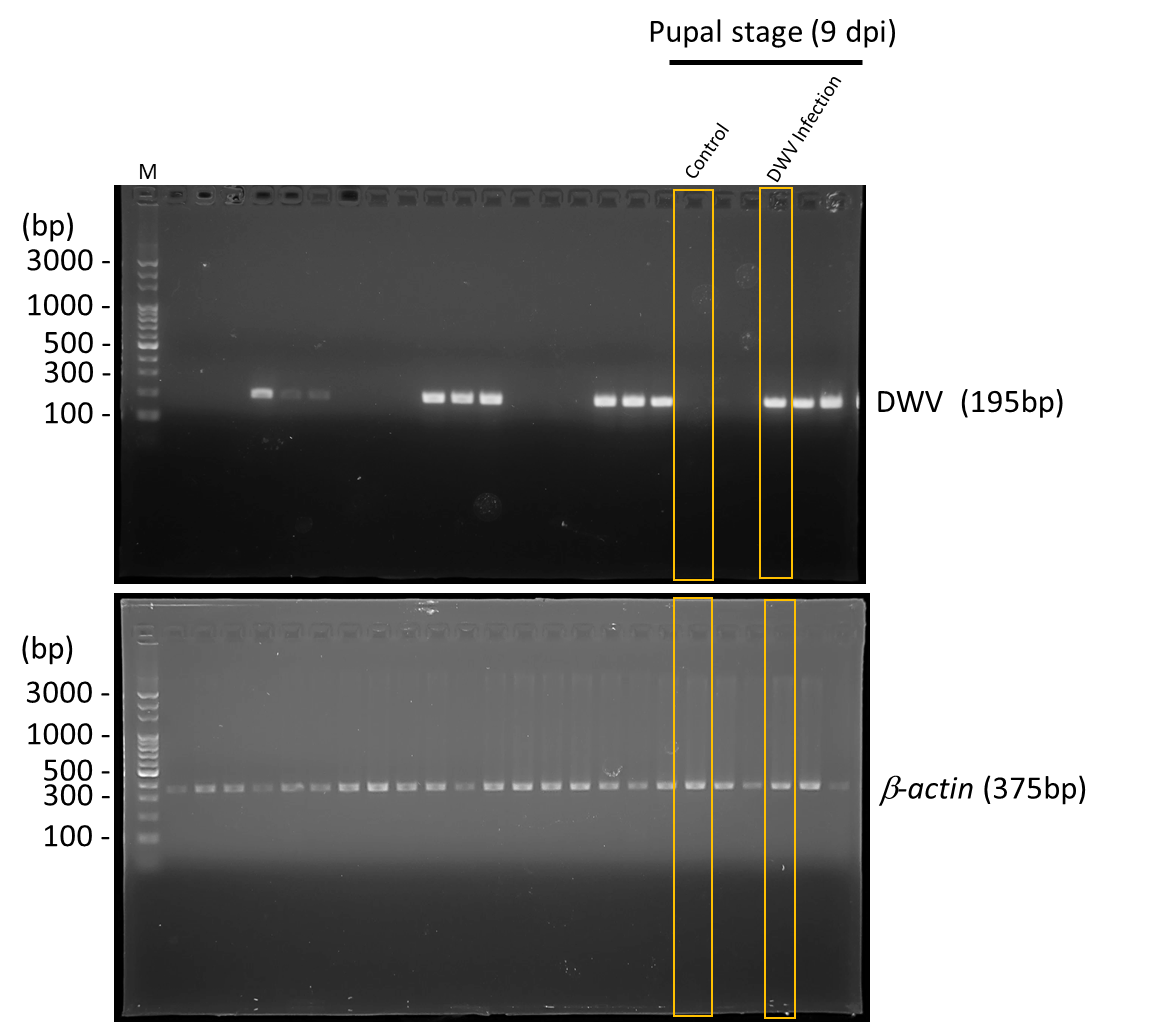


**Supplementary Figure 5.** The original DNA gel of the pupal stage was cropped from different gels (box with yellow line). These gels were re-edited in **Figure 2.**

**
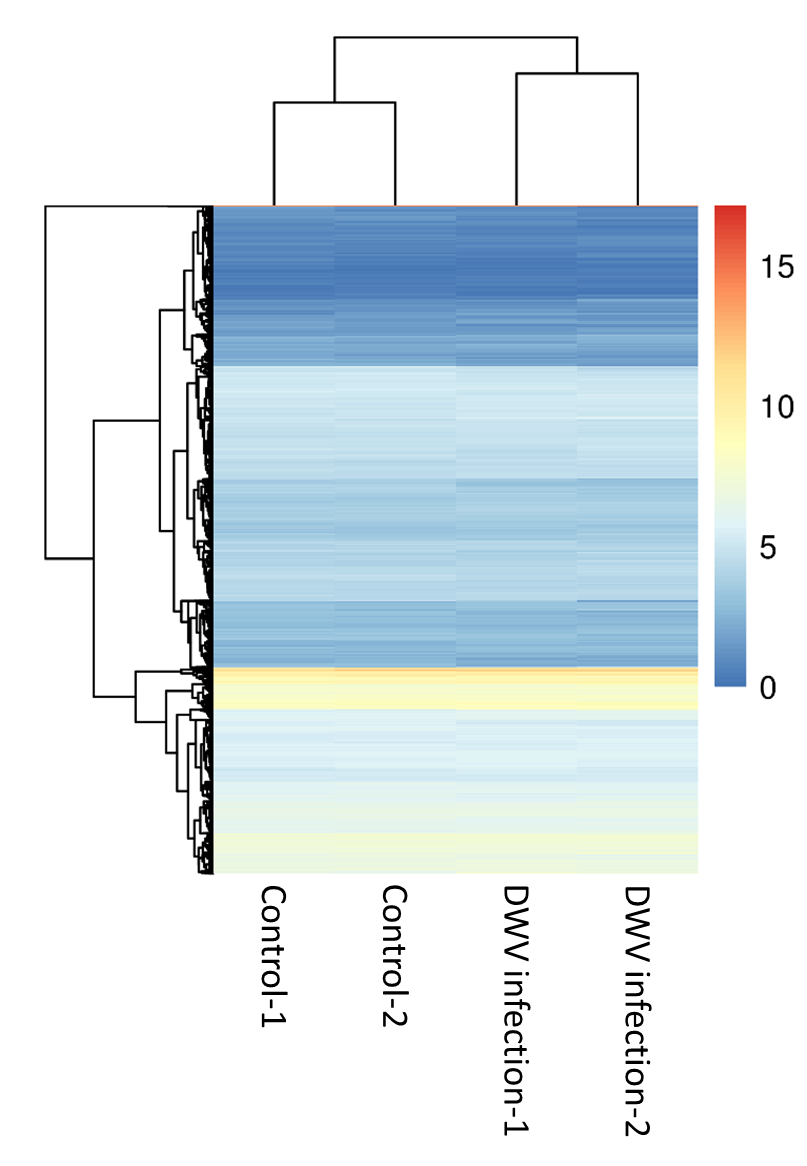
**

**Supplementary Figure 6.** The results of heat map analysis revealed a similar pattern between DWV-infected and noninfected honey bee larvae.


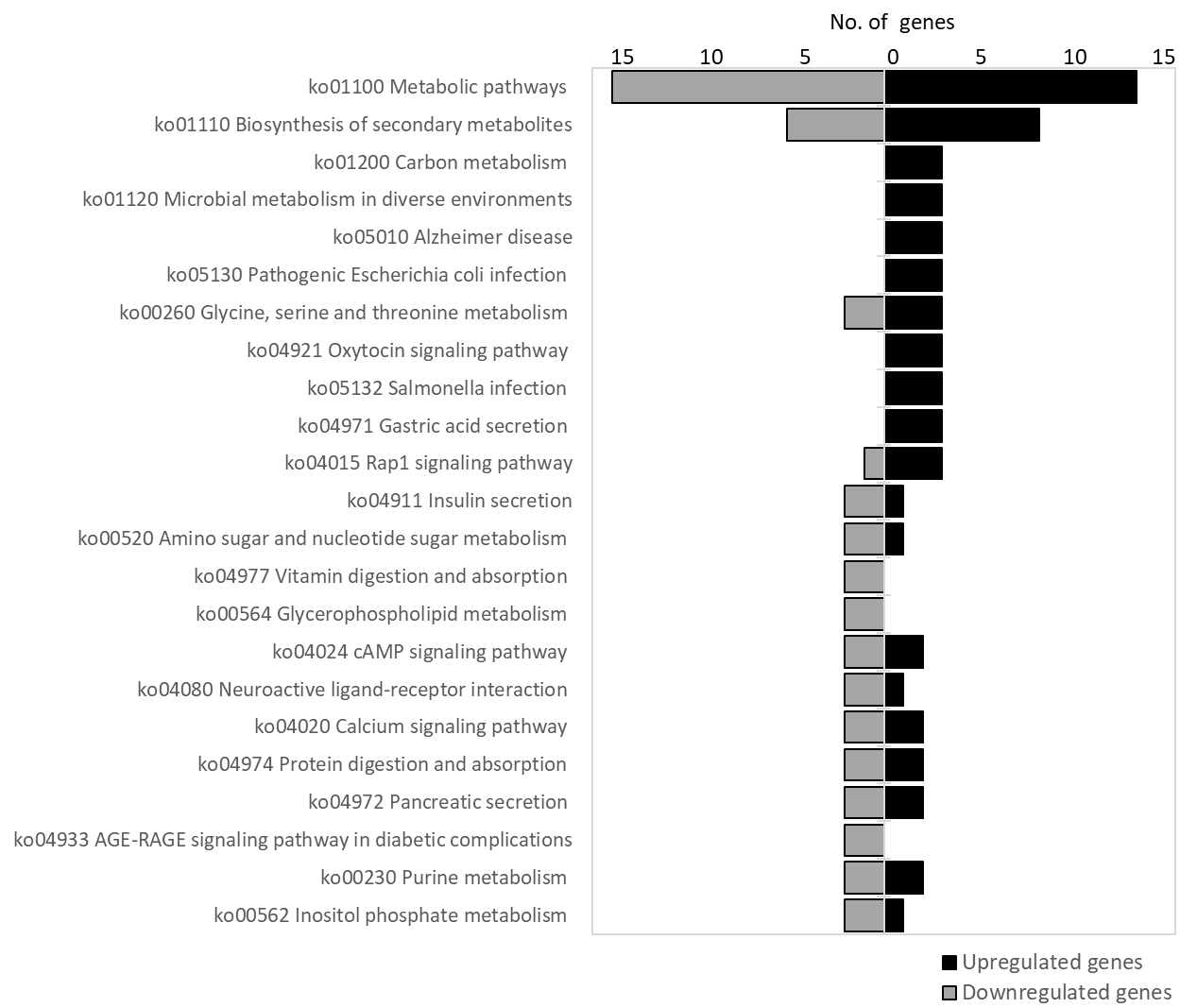


**Supplementary Figure 7.** Pathway analysis of differentially expressed honey bee genes from DWV-infected honey bee larvae using Kyoto Encyclopedia of Genes and Genomes (KEGG) pathway analysis. In total, 111 (38 upregulated genes and 73 downregulated genes) of 255 DEGs (43.53%) were annotated by KEGG pathway mapping. The top 3 pathway molecule hit numbers of DEGs in upregulated and downregulated are shown.

**Reference**

1 Benjeddou, M., Leat, N., Allsopp, M. & Davison, S. Detection of acute bee paralysis virus and black queen cell virus from honeybees by reverse transcriptase pcr. *Appl Environ Microbiol* **67**, 2384-2387, doi:10.1128/AEM.67.5.2384-2387.2001 (2001).

2 Ribière, M. *et al.* Molecular diagnosis of chronic bee paralysis virus infection. *Apidologie* **33**, 339-351 (2002).

3 Chen, Y. *et al.* Multiple virus infections in the honey bee and genome divergence of honey bee viruses. *J Invertebr Pathol* **87**, 84-93, doi:10.1016/j.jip.2004.07.005 (2004).

4 Maori, E. *et al.* Isolation and characterization of Israeli acute paralysis virus, a dicistrovirus affecting honeybees in Israel: evidence for diversity due to intra- and inter-species recombination. *J Gen Virol* **88**, 3428-3438, doi:10.1099/vir.0.83284-0 (2007).

5 Ongus, J. R. *et al.* Complete sequence of a picorna-like virus of the genus Iflavirus replicating in the mite Varroa destructor. *J Gen Virol* **85**, 3747-3755, doi:10.1099/vir.0.80470-0 (2004).
